# ﻿State of knowledge of the ladybird beetle (Coleoptera, Coccinellidae) fauna of Armenia and other Transcaucasian countries, including two new country records

**DOI:** 10.3897/zookeys.1221.127225

**Published:** 2024-12-13

**Authors:** Shoghik Ghazaryan, Marine Arakelyan, Astghik Ghazaryan, Jerzy Romanowski, Krzysztof Turlejski, Piotr Ceryngier

**Affiliations:** 1 Department of Zoology, Yerevan State University, Yerevan, Armenia; 2 Research Institute of Biology, Yerevan State University, Yerevan, Armenia; 3 Institute of Biological Sciences, Cardinal Stefan Wyszyński University, Warsaw, Poland

**Keywords:** Biodiversity, checklist, endemic species, non-native species, South Caucasus

## Abstract

Beetles (Coleoptera) have been surveyed in Armenia and other Transcaucasian countries since the first half of the 19^th^ century. Based on the literature reports and our new data, available information was gathered on the occurrence in Armenia of one of the beetle families, the ladybirds (Coccinellidae). 84 species of Coccinellidae have been reported from this country in the literature and/or collected during our recent field survey. Two of them, *Anatisocellata* (Linnaeus, 1758) and *Tytthaspissedecimpunctata* (Linnaeus, 1761), have not been reported in the literature but were present in our field samples, so they can be considered species new to Armenia, and signify new country records. In addition to the 84 species unambiguously reported from Armenia, 14 were broadly reported from larger regions that include that country (Transcaucasia, the Caucasus) or its parts (the Araks valley). The recognized Coccinellidae fauna of Armenia is slightly poorer than the faunas of other Transcaucasian countries (Azerbaijan and Georgia): there are 92 species currently known to occur in Azerbaijan and 90 species in Georgia. Interestingly, the Armenian fauna contains more Caucasian endemics (10 species) and fewer non-natives (1 species) than the faunas of Azerbaijan (4 endemics and 2 non-natives) and Georgia (6 endemics and 6 non-natives).

## ﻿Introduction

The Caucasus ecoregion, a mountainous area on the border between south-eastern Europe and western Asia, covers the North Caucasus (part of Russia), the South Caucasus or Transcaucasia (Georgia, Armenia, and Azerbaijan, plus smaller separatist areas of South Ossetia and Abkhazia), north-eastern Turkey, and north-western Iran (Zazanashvili et al. 2020). Some parts of the region, such as Colchis (western Georgia) or the southern Caspian basin (south-eastern Azerbaijan and northern Iran), were Pleistocene glacial refugia (Hewitt 1999; Tarkhnishvili et al. 2012). Due to its immense biological diversity, which is at risk of rapid decline, the Caucasus was ranked by Myers et al. (2000) among the 25 globally recognized biodiversity hotspots. Shortly afterwards, part of its area (a small portion of southern Georgia, approximately half of Armenia and the Azerbaijani enclave of Nakhchivan) was moved to the newly distinguished Irano-Anatolian hotspot (Mittermeier et al. 2004). As a result, the Caucasus ecoregion is divided between two of the 36 currently identified biodiversity hotspots (Zazanashvili et al. 2020). As Mumladze et al. (2020) point out, the region has probably been less intensively studied than many other hotspots, partly due to its tenuous political situation over the past decades.

As a country divided by the border between the Caucasus and Irano-Anatolian hotspots, Armenia is arguably an area of particularly high but as yet insufficiently recognized biodiversity. The Armenian invertebrate fauna, due to the influence of different surrounding faunas (European, Mediterranean, Irano-Turanian), as well as the diversity of landscapes and mountainous nature of the area, is rich and characterized by a high level of endemism (Kalashian et al. 2023). In this paper, we review the available data on a small part of this biodiversity, the ladybird beetles (Coccinellidae). So far, there is no paper summarizing knowledge of the ladybird fauna of Armenia, while checklists of Coccinellidae of the other Transcaucasian states, Georgia and Azerbaijan, have recently been published (Migeon and Arabuli 2022; Snegovaya and Zare Khormizi 2022). Furthermore, the ladybirds of the westernmost portion of the Russian Caucasus along the Black Sea coast were the subject of the recent, detailed study by Bieńkowski and Orlova-Bienkowskaja (2020). Hereafter, we first outline a history of coleopterological (with special reference to Coccinellidae) exploration of Armenia followed by an annotated checklist of the Armenian ladybirds based on the literature data and our unpublished records. Finally, we compare ladybird faunas of the three Transcaucasian countries, Armenia, Azerbaijan, and Georgia. We realize that most animals do not respect political boundaries, and that it would be much more biologically sound to compare the faunas of biogeographical units instead of countries. However, this would not be easy to do, given that in many cases the provided record location data is very general. Moreover, the recently published checklists for Georgian and Azerbaijani ladybirds indicate that it would be worthwhile to compile an analogous checklist for Armenia.

### ﻿A brief history of faunistic studies of Coccinellidae in Armenia and adjacent regions

While there is a lack of publications presenting detailed studies on the Coccinellidae fauna of Armenia, there is much data on this family scattered in books and papers of a broader scope (e.g., on insects or beetles of the region). Furthermore, some systematic works on Coccinellidae, such as species descriptions or taxonomic revisions, contain data from Armenia. Surveys and publications that have particularly contributed to the knowledge of the ladybird fauna of the Caucasus and Armenia are reviewed below in chronological order.

Entomological exploration of the Caucasus began in the first half of the 19^th^ century. In 1827, during the Russo-Persian War, a researcher named Szovitz travelled with the Russian army to Transcaucasia to study the flora of the region. In addition to the numerous plants he collected there until 1830, he also caught some insect specimens. On his way back from the expedition, Szovitz fell ill and subsequently died in the Georgian province of Mingrelia in August 1830 (Faldermann 1835). The insects he collected were later examined by Faldermann (1835, 1837, 1838).

Another scientific expedition to the Caucasus organized by order of the Russian Tsar took place in 1829 and 1830, with the French naturalist Édouard Ménétries (1802–1861) in charge of the zoological part. After the expedition, Ménétries (1832) published a catalogue of recorded animal species, but independently Faldermann (1835, 1837, 1838) united the data on beetles collected by Ménétries with those collected by Szovitz to compile his ‘Fauna Entomologica Trans-Caucasica’.

In 1834 and 1835, the Caucasian entomofauna (mainly Coleoptera) was surveyed by the well-known Russian entomologist Victor Ivanovich Motschulsky, who often signed his works as T. Victor (Victor 1835, 1837). Then, probably in the 1840s and early 1850s, Wagner (1852) spent several years in the Caucasus, Transcaucasia, Armenia, Kurdistan, and western Persia, studying, among other things, the fauna of the region, including beetles. Other major 19^th^ century expeditions to the Caucasus involving beetle collecting were those of Schneider and Leder in 1875–1876 (Schneider and Leder 1879) and Leder in 1878 (Leder 1879).

Apart from externally organized Caucasian expeditions, from the 1860s onwards the region was explored as part of the activities of the Caucasus Museum in Tiflis (now Tbilisi). During numerous field trips, the museum’s director Gustav Radde and his colleagues collected abundant insect material, subsequently elaborated by eminent entomologists of the time. The animals, including beetles, held in the collection of the Caucasus Museum, were catalogued by Radde (1899).

In most of the publications reporting on the results of the surveys mentioned above, the locations are given imprecisely and many records cannot be attributed to the area of any currently existing country within its present borders. Based on Ménétries’ (1832) description of his expedition, we can conclude that it took place outside of present-day Armenia (the present-day Russian Caucasus (including Dagestan and Chechnya) and Azerbaijan), but Szovitz’s records are likely to have originated there. However, Faldermann’s (1837) compilation does not indicate which of the reported species were collected by Ménétries and which by Szovitz. The area explored by Wagner’s (1852) expedition was extensive, but his report lacks any information on the location of the animals collected. On the other hand, Wagner’s list of the collected insects is very short and the family Coccinellidae is represented there by only three or four species (two of the four listed names are probably synonyms). Relatively detailed information on the location of the collected species of Coccinellidae can be found in the papers by Motschulsky (Victor 1837), Schneider and Leder (1879), Leder (1879), and Radde (1899). While Leder (1879) reported exclusively from the territories of present-day Georgia and Azerbaijan, some ladybird species reported by Motschulsky (Victor 1837) and, especially, Schneider and Leder (1879) and Radde (1899), were certainly collected within the present Armenian territory.

The first half of the 20^th^ century was a time of marked stagnation in the study of the coleopterofauna of the Caucasus. In the second half of the century, the renowned Armenian coleopterist Stepan Iablokoff-Khnzorian conducted his systematic and faunistic research, much of which was devoted to Coccinellidae (e.g., Iablokoff-Khnzorian 1971, 1972, 1974, 1982, 1983). Iablokoff-Khnzorian was primarily a taxonomist, paying little attention to providing geographical details for each of the species he recorded. Therefore, as in some of the 19^th^ century publications, the distribution data he provided often do not allow for a clear attribution to a specific country. After the period of Iablokoff-Khnzorian’s research activities, from the 1990s to the present, virtually nothing has been published on ladybirds of Armenia, with the exception of reports by Kalashian et al. (2017, 2019) on the arrival and spread of the invasive harlequin ladybird, *Harmoniaaxyridis* (Pallas, 1773), in that country.

## ﻿Checklist of Coccinellidae reported from Armenia

The checklist includes available literature reports supplemented by previously unpublished data collected between 2018 and 2023 in all 11 provinces of Armenia by MA, AG, and M. Kalashian (National Academy of Sciences, Yerevan). The new data were recorded using standard collection methods, such as a beating tray, sweep net, or direct observation. Specimens collected by MA and AG are deposited in Research Institute of Biology of Yerevan State University, and those collected by M. Kalashian, in the Scientific Center of Zoology and Hydroecology, National Academy of Science, Yerevan.

The systematic arrangement of Coccinellidae, including the sequence of tribes, used in the checklist below follows Che et al. (2021) and the nomenclature of genera and species follows Kovář (2007). To our knowledge, for all species included in this checklist, the names provided by Kovář (2007) are valid. They are given here in bold, while the primary synonyms (provided if different from the valid names) and other synonyms (only those mentioned in the checklist) are in non-bold. The genera and species within the tribes are arranged alphabetically. Names of species that are possible but not certain to occur in Armenia (only generally reported from regions encompassing this country or its parts) are in square brackets to distinguish them from those unquestionably reported from the area of present-day Armenia. For individual species, we first provide new data (if available), including the name of the province, locality, geographical coordinates, altitude, date of collection, number of specimens collected, and the collector’s name. We then list the literature reports, starting with those pointing unambiguously to Armenia and then moving on to more general location descriptions that do not exclude Armenia, such as the “Araks valley”, “Transcaucasia”, or the “Caucasus”. At present, Araks’ river source and initial course lie in Turkey, which then flows along the Turkish-Armenian border, next the border between Turkey and Nakhchivan, Iran and Nakhchivan, Iran and Armenia, and Iran and Azerbaijan. Finally, it flows to the Kura River on the Azerbaijani territory.

### ﻿Subfamily Coccinellinae Latreille, 1807

#### ﻿Tribe Stethorini Dobzhansky, 1924

***Stethorusgilvifrons* (Mulsant, 1850)**

*Scymnusgilvifrons* Mulsant, 1850

**Literature data.** Armenia: Schneider and Leder (1879) (Echmiadzin, Yerevan); Jacobson (1915); Kovář (2007). Araks valley: Iablokoff-Khnzorian (1983).

***Stethoruspusillus* (Herbst, 1797)**

*Scymnuspusillus* Herbst, 1797

**Literature data.** Armenia: Kovář (2007). Caucasus: Iablokoff-Khnzorian (1983).

#### ﻿Tribe Coccinellini Latraille, 1807

***Adaliabipunctata* (Linnaeus, 1758)**

*Coccinellabipunctata* Linnaeus, 1758

*Coccinellafasciatopunctata* Faldermann, 1835

**New data.** Kotayk: • Tsaghkadzor, 40.5314°N, 44.7249°E, 1841 m a.s.l., Jul. 2021, 5 exx. (leg. M. Arakelyan); Gegharkunik: • Akunk, 40.1572°N, 45.7263°E, 1965 m a.s.l., 19 Oct. 2020, 4 exx. (leg. M. Arakelyan); Vayots Dzor: • Hors, 39.8625°N, 45.2303°E, 1694 m a.s.l., 23 Jul. 2021, 1 ex. (leg. M. Arakelyan); Armavir: • river Kasagh, 40.1047°N, 44.2360°E, 870 m a.s.l., 1 Jun. 2021, 1 ex. (leg. M. Arakelyan); Syunik: • Lichk, 39.6074°N, 46.1113°E, 1929 m a.s.l., 17 May 2022, 8 exx. (leg. M. Arakelyan); Aragatsotn: • Kar­bi, 40.3233°N, 44.3800°E, 1303 m a.s.l., 22 Jun. 2019, 2 exx. (leg. A. Ghazaryan).

**Literature data.** Armenia: Kovář (2007). Caucasus: Heyden et al. (1891, 1906) (as *Adaliafasciatopunctatastictica* Muls.); Jacobson (1915) (as *A.fasciatopunctata* Fald.); Iablokoff-Khnzorian (1983).

***Adaliadecempunctata* (Linnaeus, 1758)**

*Coccinelladecempunctata* Linnaeus, 1758

**New data.** Kotayk: • Hankavan, 40.6019°N, 44.6185°E, 1990 m a.s.l., 25.06.2021, 2 exx. (leg. M. Arakelyan); • Tsaghkadzor, 40.5313°N, 44.7249°E, 1841 m a.s.l., July, 2021, 1 ex. (leg. M. Arakelyan); Syunik: • Shenatagh, 39.38°N, 46.1322°E, 2500 m a.s.l., 18.06.2021, 1 ex. (leg. A. Ghazaryan); • Lichk, 39.6073°N, 46.1113°E, 1929 m a.s.l., 17.05.2022, 12 exx.; • Harsnadzor rest., 39.3927°N, 46.2784°E, 1929 m a.s.l., 18.05.2022, 2 exx. (leg. M. Arakelyan).

**Literature data.** Armenia: Schneider and Leder (1879); Jacobson (1915); Kovář (2007). Caucasus: Iablokoff-Khnzorian (1982, 1983).

***Anatisocellata* (Linnaeus, 1758), new country record**

*Coccinellaocellata* Linnaeus, 1758

**New data.** Kotayk: • Tsaghkadzor, 40.5313°N, 44.7249°E, 1841 m a.s.l., July, 2021, 1 ex. (leg. M. Arakelyan).

**Literature data.** Caucasus: Iablokoff-Khnzorian (1982, 1983).

**Remarks.** Prior to the record provided above, *A.ocellata* has not been explicitly reported as occurring in Armenia.

***Anisostictacaucasica* (Fleischer, 1900)**

*Adoniaarctica* v. *caucasica* Fleischer, 1900

**Literature data.** Armenia: Jacobson (1915) (as Hippodamiaarcticasubsp.caucasica Fleisch.); Kovář (2007). Araks valley: Fleischer (1900) (as *Adoniaarctica* v. *caucasica*). Caucasus: Heyden et al. (1906) (as *Hippodamiaarctica* v. *caucasica* Fleischer), Winkler (1927) (as *H.arctica* ?s. *caucasica* Fleisch.).

**Remarks.** There is no consensus on what Fleischer’s (1900)*A.arctica* v. *caucasica* really is. While Fürsch (1977) and Kovář (2007) considered it as a distinct species belonging to the genus *Anisosticta*, according to Iablokoff-Khnzorian (1982, 1983), it is a synonym of Hippodamia (Semiadalia) schneideri (Weise, 1878). In his opinion, Fleischer’s type is a small, untypically colored specimen of H. (Semiadalia) (= *Ceratomegilla*) *schneideri*.

***Anisostictanovemdecimpunctata* (Linnaeus, 1758)**

*Coccinellanovemdecimpunctata* Linnaeus, 1758

*Anisostictaegena* Weise, 1887

**Literature data.** Armenia: Iablokoff-Khnzorian (1983) (whole Caucasus); Kovář (2007). Caucasus: Heyden et al. (1891) (as *A. 19-punctata* v. *egena* Ws.); Heyden et al. (1906) (as *A.egena* Ws.).

***Aphidectaobliterata* (Linnaeus, 1758)**

*Coccinellaobliterata* Linnaeus, 1758

**Literature data.** Armenia: Kovář (2007). Caucasus: Winkler (1927).

***Bulaealichatschovii* (Hummel, 1827)**

*Coccinella Lichatschovii* Hummel, 1827

**Literature data.** Armenia: Schneider and Leder (1879) (Echmiadzin); Jacobson (1915); Iablokoff-Khnzorian (1983) (whole Caucasus); Kovář (2007). Araks valley: Radde (1899) (as *B. Lichatschovi* var. *coronata* Weise.). Transcaucasia: Faldermann (1837). Caucasus: Weise (1879, 1885) (as *B. Lichatschovii* v. *coronata* Ws.); Heyden et al. (1891, 1906) (as *B. Lichatschovi* v. *coronata* Ws.).

[***Bulaealividulabocandei* Mulsant, 1850**]

*Bulaea Bocandei* Mulsant, 1850

**Literature data.** Caucasus: Weise (1885) (as *B. Lichatschovii* v. *pallida* Motsch.).

**Remark.**Biranvand et al. (2024) consider *B.lividulabocandei* a separate species, *B.bocandei* Mulsant, 1850.

***Calviadecemguttata* (Linnaeus, 1767)**

*Coccinelladecemguttata* Linnaeus, 1767

*Calviahololeuca* Mulsant, 1850 (synonymized by Iablokoff-Khnzorian (1972))

**Literature data.** Armenia: Kovář (2007). Caucasus: Mulsant (1850, 1866) (as *Calviahololeuca*); Weise (1879, 1885) (as *Halyziadecemguttata* v. *hololeuca* Muls.); Heyden et al. (1891) (as *Halyziadecemguttata* v. *hololeuca* Muls.); Heyden et al. (1906) (as *Calvia 10-guttata* v. *hololeuca* Muls.); Iablokoff-Khnzorian (1972).

***Calviaquatuordecimguttata* (Linnaeus, 1758)**

*Coccinellaquatuordecimguttata* Linnaeus, 1758

*Propylaea Rosti* Weise, 1891

**New data.** Kotayk: • Tsaghkadzor, 40.5313°N, 44.7249°E, 1841 m a.s.l., July, 2021, 2 exx. (leg. M. Arakelyan); Syunik: • Lichk, 39.6073°N, 46.1113°E, 1929 m a.s.l., 17.05.2022, 1 ex. (leg. M. Arakelyan).

**Literature data.** Armenia: Kovář (2007). Caucasus: Heyden et al. (1906) (as *Propylaea Rosti* Ws.); Jacobson (1915) (as *Propylaea Rosti* Ws.); Winkler (1927) (as *Propylaea Rosti* Ws.).

[***Calviaquindecimguttata* (Fabricius, 1777)**]

*Coccinellaquindecimguttata* Fabricius, 1777

**Literature data.** Caucasus: Iablokoff-Khnzorian (1982, 1983).

***Ceratomegillaapicalis* (Weise, 1879)**

*Adaliaapicalis* Weise, 1879

**Literature data.** Armenia: Iablokoff-Khnzorian (1982, 1983); Kovář (2007). Caucasus: Weise (1885); Heyden et al. (1891, 1906); Winkler (1927).

***Ceratomegillanotata* (Laicharting, 1781)**

*Coccinellanotata* Laitcharting, 1781

**Literature data.** Armenia: Kovář (2007). Caucasus: Heyden et al. (1906); Winkler (1927).

***Ceratomegillaschelkovnikovi* (Dobzhansky, 1927)**

*Semiadaliashelkovnikovi* Dobzhansky, 1927

**Literature data.** Armenia: Dobzhansky (1927) (lake Sevan, Yerevan); Iablokoff-Khnzorian (1983); Kovář (2007).

**Remarks.** Dobzhansky’s original spelling of the specific epithet is ‘*shelkovnikovi*’. Type locality: lake Sevan (Goktsha-See) in Armenia.

***Ceratomegillaschneideri* (Weise, 1878)**

*Coccinella Schneideri* Weise, 1878

**Literature data.** Armenia: Schneider and Leder (1879) (Alexandrapol (=Gyumri)); Jacobson (1915); Iablokoff-Khnzorian (1983); Kovář (2007). Caucasus: Weise (1879) (as *Adalia Schneideri* Ws.); Weise (1885); Heyden et al. (1891, 1906); Winkler (1927).

***Ceratomegillaundecimnotata* (Schneider, 1792)**

*Coccinellaundecimnotata* Schneider, 1792

*Coccinella Saliana* Faldermann, 1837

*Coccinellamaritima* Ménétries, 1832

**Literature data.** Armenia: Iablokoff-Khnzorian (1982); Kovář (2007); Ceryngier et al. (2023) (Tigranashen, as a host of a hymenopterous parasitoid *Dinocampuscoccinellae* (Schrank)). Transcaucasia: Faldermann (1837) (as *Coccinella Saliana* Fald. and *C.maritima* Ménétries). Caucasus: Weise (1879) (as *Adaliaundecimnotata* Schneid.); Weise (1885); Radde (1899).

***Coccinellaalpigrada* (Iablokoff-Khnzorian, 1957)**

*Adaliaalpigrada* Iablokoff-Khnzorian, 1957

**Literature data.** Armenia: Iablokoff-Khnzorian (1957, 1982, 1983); Kovář (2007).

**Remarks.** Described from Armenia. Type locality: Yanykh, Martuni region (province Gegharkunik).

***Coccinellamagnifica* Redtenbacher, 1843**

*Coccinelladistincta* Faldermann, 1837

**New data.** Kotayk: • Arzakan, 40.4494°N, 44.6063°E, 1489 m a.s.l., 07.06.2021, 5 exx. (leg. M. Arakelyan).

**Literature data.** Armenia: Kovář (2007). Transcaucasia: Faldermann (1837) (as *C.distincta*); Iablokoff-Khnzorian (1983). Caucasus: Weise (1885) (as *C.distincta* Faldermann); Heyden et al. (1891, 1906) (as *C.distincta* Fald.); Winkler (1927) (as *C.divaricata* Ol.).

***Coccinellaquinquepunctata* Linnaeus, 1758**

*Coccinellatripunctata* Rossi, 1790

**Literature data.** Armenia: Victor (1837) (as *Coccinella 3 punctata* Rossi); Mulsant (1866) (as *Coccinellatripunctata* Rossi), Kovář (2007).

**Remarks.**Kovář (2007) considers *Coccinellatripunctata* Rossi, 1790 a synonym of *C.quinquepunctata*. However, according to Crotch (1874), *C.tripunctata* Rossi is a variety of *Coccinellaundecimpunctata* L. Iablokoff-Khnzorian (1983) states that *C.quinquepunctata* is present in the Caucasus, but not in Armenia.

***Coccinellaseptempunctata* Linnaeus, 1758**

**New data.** Syunik: • Shenatagh, 39.38°N, 46.1322°E, 2500 m a.s.l., 18.06.2021, 5 exx. (leg. A. Ghazaryan); • Kajaran, 39.1511°N, 46.16°E, 1950 m a.s.l., 13.06.2021, 14 exx. (leg. A. Ghazaryan); • Zvaravank Monastery, 39.0472°N, 46.1694°E, 1815 m a.s.l., 16.05.2022, 1 ex. (leg. M. Arakelyan); • Meghri, 38.9029°N, 46.2445°E, 610 m a.s.l., 15.05.2022, 4 exx. (leg. M. Arakelyan); • Lichk, 39.6073°N, 46.1113°E, 1929 m

a.s.l., 17.05.2022, 1 ex. (leg. M. Arakelyan); Ararat: • Azat reservoir, 40.07138°N, 44.6161°E, 1025 m a.s.l., 20.09.2021, 1 ex. (leg. M. Arakelyan); • Khosrov, 40.0458°N, 44.8982°E, 1465 m a.s.l., 28.05.2021, 2 ex. (leg. M. Arakelyan); Lori: • Mets Parni, 40.8372°N, 44.1091°E, 1680 m a.s.l., 05.06.2020, 22.06.2020, 2 ex. (leg. M. Arake­lyan); Vayots Dzor: • Hors, 39.8625°N, 45.2302°E, 1694 m a.s.l., 12.06.2021, 1 ex. (leg. M. Arakelyan); • Horbategh, 39.8902°N, 45.3541°E, 1850 m a.s.l., 04.06.2023, 3 exx. (leg. M. Arakelyan); Tavush: • Voskepar, 41.0647°N, 45.0575°E, 850 m a.s.l., 26.09.2020, 1 ex. (leg. M. Arakelyan); Gegharkunik: • Dzoragyugh, 40.1694°N, 45.1986°E, 2003 m a.s.l., 06.06.2021, 1 ex. (leg. M. Arakelyan); Kotayk: • Tsagh­kadzor, 40.5313°N, 44.7249°E, 1841 m a.s.l., June 2023, 3 exx. (leg. M. Arakelyan).

**Literature data.** Armenia: Jacobson (1915) (the whole Caucasus); Kovář (2007). Caucasus: Victor (1837).

***Coccinellaundecimpunctata* Linnaeus, 1758**

**Literature data.** Armenia: Radde (1899) (Meghri); Jacobson (1915).

***Coccinulaquatuordecimpustulata* (Linnaeus, 1758)**

*Coccinellaquatuordecimpustulata* Linnaeus, 1758

**New data.** Lori: • Mets Parni, 40.8372°N, 44.1091°E, 1680 m a.s.l., 22.08.2018, 9 exx. (leg. M. Arakelyan); Syunik: • Lichk, 39.6073°N, 46.1113°E, 1929 m a.s.l., 17.05.2022, 1 ex. (leg. M. Arakelyan); Kotayk: • Gegard, 40.1553°N, 44.7913°E, 1759 m a.s.l., 22.05.2022, 1 ex. (leg. M. Arakelyan); • Tsaghkadzor, 40.5313°N, 44.7249°E, 1841 m a.s.l., June 2023, 2 exx. (leg. M. Arakelyan); Ararat: • Khosrov, 40.0458°N, 44.8982°E, 1465 m a.s.l., 28.05.2021, 2 exx. (leg. M. Arakelyan); Vayots Dzor: • Hors, 39.8625°N, 45.2302°E, 1694 m a.s.l., 12.06.2021, 1 ex. (leg. M. Arakelyan).

**Literature data.** Armenia: Radde (1899) (Helenowka (= Sevan)); Jacobson (1915); Iablokoff-Khnzorian (1982, 1983); Kovář (2007).

***Coccinulasinuatomarginata* (Faldermann, 1837)**

*Coccinellasinuato-marginata* Faldermann, 1837

**New data.** Syunik: • Lichk, 39.6073°N, 46.1113°E, 1929 m a.s.l., 17.05.2022, 1 ex. (leg. M. Arakelyan).

**Literature data.** Armenia: Jacobson (1915); Iablokoff-Khnzorian (1982, 1983); Kovář (2007). Transcaucasia: Faldermann (1837) (as *Coccinellasinuatomarginata*). Caucasus: Mulsant (1850, 1866) (as *Coccinellasinuato-marginata* Faldermann); Weise (1879, 1885); Heyden et al. (1906) (as *Synharmoniasinuatomarginata* Fald.).

***Halyziasedecimguttata* (Linnaeus, 1758)**

*Coccinellasedecimguttata* Linnaeus, 1758

**New data.** Kotayk: • Tsaghkadzor, 40.5313°N, 44.7249°E, 1841 m a.s.l., July 2021, 1 ex. (leg. M. Arakelyan).

**Literature data.** Armenia: Iablokoff-Khnzorian (1982); Kovář (2007). Caucasus: Winkler (1927); Iablokoff-Khnzorian (1983).

***Harmoniaaxyridis* (Pallas, 1773)**

*Coccinellaaxyridis* Pallas, 1773

**New data.** Kotayk: • Tsaghkadzor, 40.5313°N, 44.7249°E, 1841 m a.s.l., July 2021, 2 exx. (leg. M. Arakelyan); • Hankavan, 40.6019°N, 44.6185°E, 1990 m a.s.l., 25.06.2021, 1 ex. (leg. M. Arakelyan); Shirak: • Gyumri, 40.7942°N, 43.8452°E, 1509 m a.s.l., 15.10.2020, 1 ex. (leg. M. Arakelyan); Yerevan: • Yerevan State University, 40.1817°N, 44.5261°E, 990 m a.s.l., 27.07.2020, 1 ex. (leg. M. Arakel­yan); Aragatsotn: • Karbi, 40.3233°N, 44.3800°E, 1303 m a.s.l., 22.06.2019, 1 ex. (A.Ghazaryan); Lori: • Amrakits, 40.0002°N, 44.4303°E, 1380 m a.s.l., 31.05.2023, 2 exx. (leg. M. Arakelyan); • Pushkino, 40.9688°N, 44.4144°E, 1450 m a.s.l., 26.06.2023, 1 ex. (leg. M. Arakelyan); • Sanahin, 41.0873°N, 44.6661°E, 1016 m a.s.l., 28.05.2023, 1 ex. (leg. M. Arakelyan); • Privolnoye, 41.1709°N, 44.4415°E, 1629 m a.s.l., 14.06.2020, 1 ex. (leg. M. Kalashian); • Gyulagarak, 40.9620°N, 44.4696°E, 1358 m a.s.l., 09.07.2020, 15.07.2020, 20.07.2020, 3 exx. (leg. M. Kalashian); • Kachachkut, 41.1600°N, 44.5852°E, 2510 m a.s.l., 18.07.2021, 1 ex. (leg. M. Kalashian); • Margahovit, 40.70933°N, 44.66645°E, 1918 m a.s.l., 04.07.2021, 1 ex. (leg. M. Kalashian); • Odzun, 41.038577°N, 44.627781°E, 1034 m a.s.l., 30.05.2021, 1 ex. (leg. M. Kalashian); Gegharkunik: • Aygut, 40.6878°N, 45.1473°E, 1268 m a.s.l., 18.07.2020, 1 ex. (leg. M. Kalashian); • Shorzha, 40.49845°N, 45.29701°E, 1938 m a.s.l., 12.07.2021, 1 ex. (leg. M. Ka­lashian); Ararat: • Sipanik, 40.0796°N, 44.3637°E, 843 m a.s.l., 08.09.2020, 1 ex. (leg. M. Kalashian); • Armash, 39.796°N, 44.8415°E, 1203 m a.s.l., 07.08.2021, 1 ex. (leg. M. Kalashian); Tavush: • Gosh, 40.7409°N, 45.0334°E, 1080 m a.s.l., 18.06.2021, 1 ex. (leg. M. Kalashian).

**Literature data.** Armenia: Kalashian et al. (2017, 2019).

***Hippodamiatredecimpunctata* (Linnaeus, 1758)**

*Coccinellatredecimpunctata* Linnaeus, 1758

*Hippodamiasignata* Faldermann, 1837

**New data.** Vayots Dzor: • Spitakavor church, 39.8297°N, 45.3644°E, 540 m a.s.l., 12.08.2020, 1 ex. (leg. M. Arakelyan).

**Literature data.** Armenia: Jacobson (1915) (as H.tredecimpunctatasubsp.signata Fald.) (Yerevan); Kovář (2007). Transcaucasia: Faldermann (1837) (as *H.signata* Fald.). Caucasus: Weise (1885); Heyden et al. (1891, 1906); Winkler (1927); Iablokoff-Khnzorian (1982, 1983).

***Hippodamiavariegata* (Goeze, 1777)**

*Coccinellavariegata* Goeze, 1777

**New data.** Gegharkunik: • Tsovak, 40.1819°N, 45.635°E, 1920 m a.s.l., 04.06.2021, 2 exx. (leg. M. Arakelyan); Ararat: • Ranchpar, 40.0253°N, 44.3703°E, 834 m a.s.l., 05.06.2021, 1 ex. (leg. M. Arakelyan); • Khosrov, 40.0458°N, 44.8982°E, 1465 m a.s.l., 28.05.2021, 1 ex. (leg. M. Arakelyan); Vayots Dzor: • Hors, 39.8625°N, 45.2302°E, 1694 m a.s.l., 12.06.2021, 2 exx. (leg. M. Arakel­yan); Kotayk: • Hankavan, 40.6019°N, 44.6185°E, 1990 m a.s.l., 25.06.2021, 1 ex. (leg. M. Arakelyan); Tavush: • Voskepar, 41.0647°N, 45.0575°E, 850 m a.s.l., 26.09.2020, 2 exx. (leg. M. Arakelyan).

**Literature data.** Armenia: Radde (1899) (Echmiadzin); Jacobson (1915) (Yerevan); Kovář (2007); Ceryngier et al. (2023) (Nzhdeh, as a host of *D.coccinellae*).

***Myrrhaoctodecimguttata* (Linnaeus, 1758)**

*Coccinellaoctodecimguttata* Linnaeus, 1758

**Literature data.** Armenia: Iablokoff-Khnzorian (1983); Kovář (2007).

***Myziaoblongoguttata* (Linnaeus, 1758)**

*Coccinellaoblongoguttata* Linnaeus, 1758

**Literature data.** Armenia: Iablokoff-Khnzorian (1982, 1983). Caucasus: Winkler (1927) (as *Paramysiaoblongoguttata* L.).

***Oenopiaconglobata* (Linnaeus, 1758)**

*Coccinellaconglobata* Linnaeus, 1758

**New data.** Gegharkunik: • Akunk, 40.1572°N, 45.7263°E, 1965 m a.s.l., 19.10.2020, 3 exx. (leg. M. Arakelyan); Ararat: • Ranchpar, 40.0252°N, 44.3702°E, 834 m a.s.l., 05.06.2021, 1 ex. (leg. M. Arakelyan); Kotayk: • Tsaghkadzor, 40.5313°N, 44.7249°E, 1841 m a.s.l., July 2021, 1 ex. (leg. M. Arakelyan); Ar­mavir: • river Kasagh, 40.1046°N, 44.2359°E, 870 m a.s.l., 01.06.2021, 1 ex. (leg. M. Arakelyan); Syunik: • Lichk, 39.6073°N, 46.1113°E, 1929 m a.s.l., 17.05.2022, 2 exx. (leg. M. Arakelyan).

**Literature data.** Armenia: Iablokoff-Khnzorian (1972); Kovář (2007). Transcaucasia: Iablokoff-Khnzorian (1983).

***Oenopiaimpustulata* (Linnaeus, 1767)**

*Coccinellaimpustulata* Linnaeus, 1767

*Coccinellacaucasica* Motschulsky, 1837 (synonymized by Kovář (2007))

**Literature data.** Armenia: Kovář (2007). Transcaucasia: Iablokoff-Khnzorian (1983). Caucasus: Victor (1837) (as *Coccinellacaucasica*); Mulsant (1850) (as *Harmoniacaucasica* Motschoulsky); Heyden et al. (1891) (as *Harmoniaconglobata* v. *caucasica* Motsch.); Heyden et al. (1906) (as *Synharmoniaconglobata* v. *caucasica* Motsch.); Iablokoff-Khnzorian (1972).

***Oenopialynceaagnatha* (Rosenhauer, 1847)**

*Coccinellalynceaagnatha* Rosenhauer, 1847

**Literature data.** Armenia: Iablokoff-Khnzorian (1982); Kovář (2007). Transcaucasia: Iablokoff-Khnzorian (1983).

***Oenopiaoncina* (Olivier, 1808)**

*Coccinellaoncina* Olivier, 1808

*Coccinellaasiatica* Weise, 1885

*Coccinellapersica* Faldermann, 1837

**Literature data.** Armenia: Schneider and Leder (1879) (as *Coccinellapersica* Faldermann) (Echmiadzin, Tarstschai); Weise (1885) (as *Harmonialyncea* v. *asiatica* Ws. and *H.lyncea* v. *persica* Faldermann); Jacobson (1915) (Yerevan); Kovář (2007). Transcaucasia: Faldermann (1837) (as *Coccinellapersica* Faldermann); Heyden et al. (1891) (as *Harmonialyncea* v. *asiatica* Ws.); Heyden et al. (1906) (as Synharmoniaoncinaa.asiatica Ws.); Iablokoff-Khnzorian (1983).

***Propyleaquatuordecimpunctata* (Linnaeus, 1758)**

*Coccinellaquatuordecimpunctata* Linnaeus, 1758

**New data.** Armavir: • river Kasagh, 40.1046°N, 44.2359°E, 870 m a.s.l., 01.06.2021, 2 exx. (leg. M. Arakelyan); Ararat: • Khosrov, 40.0458°N, 44.8982°E, 1465 m a.s.l., 28.05.2021, 3 exx. (leg. M. Arakelyan); Syunik: • Lichk, 39.6073°N, 46.1113°E, 1929 m a.s.l., 17.05.2022, 3 exx. (leg. M. Arakelyan); Aragatsotn: • Karbi, 40.3233°N, 44.3800°E, 1303 m a.s.l., 22.06.2019, 6 exx. (leg. M. Arakelyan); Lori: • Mets Parni, 40.8372°N, 44.1091°E, 1680 m a.s.l., 22.08.2018, 1 ex. (leg. M. Arakelyan); Kotayk: • Tsagh­kadzor, 40.5313°N, 44.7249°E, 1841 m a.s.l., June 2023, 3 exx. (leg. M. Arakelyan).

**Literature data.** Armenia: Radde (1899) (as *Halyziaquatuordecimpunctata* L.) (Yerevan); Jacobson (1915) (Yerevan); Kovář (2007); Ceryngier et al. (2023) (Khor Virap, as a host of *D.coccinellae*). Caucasus: Winkler (1927).

***Psylloboravigintiduopunctata* (Linnaeus, 1758)**

*Coccinellavigintiduopunctata* Linnaeus, 1758

**New data.** Kotayk: • Tsaghkadzor, 40.5313°N, 44.7249°E, 1841 m a.s.l., July 2021, 1 ex., June 2023, 5 exx. (leg. M. Arakelyan); Syunik: • Lichk, 39.6073°N, 46.1113°E, 1929 m a.s.l., 17.05.2022, 2 exx. (leg. M. Arakelyan); Lori: • Mets Parni, 40.8372°N, 44.1091°E, 1680 m a.s.l., 22.08.2018, 3 exx. (leg. M. Arakelyan); Aragatsotn: • Kar­bi, 40.3233°N, 44.3800°E, 1303 m a.s.l., 22.06.2019, 7 exx. (leg. M. Arakelyan).

**Literature data.** Armenia: Schneider and Leder (1879) (Echmiadzin, Yerevan); Radde (1899) (as *Halyziavigintiduopunctata* L.) (Yerevan); Jacobson (1915) (Yerevan); Kovář (2007).

[***Tytthaspisgebleri* (Mulsant, 1850)**]

*Micraspisgebleri* Mulsant, 1850

*Coccinellalineola* Gebler, 1843

**Literature data.** Caucasus: Winkler (1927) (as *Tytthaspislineola* Gebl.).

***Tytthaspissedecimpunctata* (Linnaeus, 1761), new country record**

*Coccinellasedecimpunctata* Linnaeus, 1761

**New data.** Lori: • Mets Parni, 40.8372°N, 44.1091°E, 1680 m a.s.l., 22.08.2018, 2 exx. (leg. M. Arakelyan); Syunik: • Lichk, 39.6073°N, 46.1113°E, 1929 m a.s.l., 17.05.2022, 1 ex. (leg. M. Arakelyan).

**Remark.** To the best of our knowledge, *T.sedecimpunctata* has not previously been reported from Armenia.

***Vibidiaduodecimguttata* (Poda von Neuhaus, 1761)**

*Coccinelladuodecimguttata* Poda von Neuhaus, 1761

**New data.** Vayots Dzor: • Hors, 39.8625°N, 45.2302°E, 1694 m a.s.l., 23.07.2021, 1 ex. (leg. M. Arakelyan).

**Literature data.** Armenia: Kovář (2007). Transcaucasia: Iablokoff-Khnzorian (1983).

#### ﻿Tribe Epilachnini Mulsant, 1846

***Henosepilachnaargus* (Geoffroy, 1785)**

*Coccinellaargus* Geoffroy, 1785

**Literature data.** Armenia: Iablokoff-Khnzorian (1980, 1981, 1983) (Araks valley in Armenia); Kovář (2007). Caucasus: Radde (1899); Jacobson (1915).

***Subcoccinellavigintiquatuorpunctata* (Linnaeus, 1758)**

*Coccinellavigintiquatuorpunctata* Linnaeus, 1758

*Coccinellacolchica* Motschulsky, 1839

**New data.** Kotayk: • Arzakan, 40.4494°N, 44.6063°E, 1489 m a.s.l., 07.06.2021, 1 ex. (leg. M. Arakelyan).

**Literature data.** Armenia: Schneider and Leder (1879) (Alexandrapol (=Gyumri)); Jacobson (1915) (Yerevan); Iablokoff-Khnzorian (1980, 1983); Kovář (2007). Caucasus: Mulsant (1850) (as *Epilachnacolchica* Motschoulsky); Heyden et al. (1906); Winkler (1927).

#### ﻿Tribe Scymnini Mulsant, 1846

***Clitostethusarcuatus* (Rossi, 1794)**

*Coccinellaarcuata* Rossi, 1794

**Literature data.** Armenia: Iablokoff-Khnzorian (1983) (in the Caucasus reaches the Araks valley); Kovář (2007). Caucasus: Winkler (1927).

[**Nephus (Bipunctatus) bipunctatus (Kugelann, 1794)**]

*Scymnusbipunctatus* Kugelann, 1794

**Literature data.** Transcaucasia: Iablokoff-Khnzorian (1983).

**Nephus (Nephus) ludyi (Weise, 1879)**

*Scymnusludyi* Weise, 1879

*Nephusponticus* Iablokoff-Khnzorian, 1970

**Literature data.** Armenia: Iablokoff-Khnzorian (1970a, 1983) (as *N.ponticus* Iablokoff-Khnzorian, 1970) (Yerevan); Kovář (2007).

**Nephus (Nephus) quadrimaculatus (Herbst, 1783)**

*Sphaeridiumquadrimaculatum* Herbst, 1783

**Literature data.** Armenia: Iablokoff-Khnzorian (1983) (N Armenia, Yerevan).

**Nephus (Sidis) caucasicus (Weise, 1929)**

*Scymnuscaucasicus* Weise, 1929

*Scymnusplagiatus* Weise, 1878

**Literature data.** Armenia: Schneider and Leder (1879) (as *Scymnusplagiatus* Weise nov. sp.) (Yerevan); Weise (1879, 1885) (as Scymnus (Nephus) plagiatus Ws.) (Yerevan); Jacobson (1915) (as *N.plagiatus* Ws.) (Yerevan); Iablokoff-Khnzorian (1983) Yerevan and its vicinity); Kovář (2007). Caucasus: Heyden et al. (1891, 1906) (as *N.plagiatus*).

***Scymniscusbiflammulatus* (Motschulsky, 1837)**

*Scymnusbiflammulatus* Motschulsky, 1837

**Literature data.** Armenia: Iablokoff-Khnzorian (1983) (almost whole Caucasus); Kovář (2007). Caucasus: Weise (1879, 1885) (as *Scymnusbiflammulatus* Motsch.); Heyden et al. (1891, 1906) (as *Sidisbiflammulatus* Motsch.); Jacobson (1915) (as *Sidisbiflammulatus* Motsch.); Winkler (1927) (as *Sidisbiflammulatus* Mtsch.).

***Scymniscusbiguttatus* (Mulsant, 1850)**

*Scymnusbiguttatus* Mulsant, 1850

*Scymnusbipustulatus* Motschulsky, 1837

**Literature data.** Armenia: Jacobson (1915) (as *Sidisbiguttatus* Muls.) (Yerevan). Araks valley: Heyden et al. (1906) (as *Sidisbiguttatus* a. *4-guttatus* Fleisch). Caucasus: Weise (1879) (as Scymnus (Sidis) bipustulatus Motsch.); Weise (1885) (as Scymnus (Sidis) biguttatus Muls.); Winkler (1927) (as *Sidisbiguttatus* Muls.).

**Scymnus (Mimopullus) pharaonis Motschulsky, 1851**

*Scymnuspharaonis* Motschulsky, 1851

Scymnus (Pullus) araraticus Iablokoff-Khnzorian, 1969

**Literature data.** Armenia: Iablokoff-Khnzorian (1969, 1972, 1983) (as Scymnus (Pullus) araraticus Iablokoff-Khnzorian, 1969) (Kapan region, Yerevan vicinity); Kovář (2007).

**Scymnus (Neopullus) haemorrhoidalis Herbst, 1797**

*Scymnushaemorrhoidalis* Herbst, 1797

**Literature data.** Armenia: Schneider and Leder (1879) (Yerevan); Jacobson (1915) (Yerevan); Iablokoff-Khnzorian (1983) (whole Caucasus); Kovář (2007).

**Scymnus (Neopullus) limbatus Stephens, 1832**

*Scymnuslimbatus* Stephens, 1832

**Literature data.** Armenia: Iablokoff-Khnzorian (1972, 1983) (Yerevan); Kovář (2007).

**Scymnus (Neopullus) testaceus Motschulsky, 1837**

*Scymnustestaceus* Motschulsky, 1837

**Literature data.** Armenia: Iablokoff-Khnzorian (1972, 1983) (whole Caucasus). Caucasus: Weise (1879); Heyden et al. (1891, 1906); Radde (1899).

**Scymnus (Pullus) argutus Mulsant, 1850**

*Scymnusargutus* Mulsant, 1850

**Literature data.** Armenia: Mulsant (1850); Weise (1885); Heyden et al. (1891); Iablokoff-Khnzorian (1972); Kovář (2007). Araks valley: Iablokoff-Khnzorian (1983). Caucasus: Winkler (1927).

**Remark.** Described from Armenia without specifying exact locality (l’Arménie (collect. Motschoulsky)) (Mulsant 1850).

[**Scymnus (Pullus) auritus Thunberg, 1795**]

*Scymnusauritus* Thunberg, 1795

**Literature data.** Caucasus: Winkler (1927).

**Scymnus (Pullus) fraxini Mulsant, 1850**

*Scymnusfraxini* Mulsant, 1850

**Literature data.** Armenia: Kovář (2007). Caucasus: Mulsant (1850); Heyden et al. (1891, 1906); Winkler (1927).

**Scymnus (Pullus) subvillosus (Goeze, 1777)**

*Coccinellasubvillosa* Goeze, 1777

**Literature data.** Armenia: Schneider and Leder (1879) (Yerevan); Jacobson (1915) (Yerevan); Kovář (2007). Caucasus: Heyden et al. (1891); Iablokoff-Khnzorian (1983).

**Scymnus (Pullus) suturalis Thunberg, 1795**

*Scymnussuturalis* Thunberg, 1795

**Literature data.** Armenia: Kovář (2007). Caucasus: Iablokoff-Khnzorian (1983).

**Scymnus (Scymnus) apetzi Mulsant, 1846**

*Scymnus Apetzii* Mulsant, 1846

*Scymnusstigmatopterus* Faldermann, 1837 (synonymized by Fürsch et al. (1967))

*Scymnuscorpulentus* Mulsant, 1850 (synonymized by Fürsch et al. (1967))

**Literature data.** Armenia: Schneider and Leder (1879) (Echmiadzin, Yerevan); Radde (1899) (Yerevan); Jacobson (1915) (Yerevan); Fürsch et al. (1967) (lectotype of *S.stigmatopterus* Fald.) (Sadaraki); Iablokoff-Khnzorian (1983) (in Transcaucasia the most common *Scymnus* species); Kovář (2007). Transcaucasia: Faldermann (1837) (as *S.stigmatopterus* Fald.); Mulsant (1850) (as *S.corpulentus*). Caucasus: Heyden et al. (1891) (as *S.corpulentus* Muls.).

**Remark.**Kovář (2007) places *S.stigmatopterus* among the taxa incertae sedis.

**Scymnus (Scymnus) flavicollis Redtenbacher, 1843**

*Scymnusfrontalis* v. *araxicola* Fleischer, 1900

**Literature data.** Armenia: Kovář (2007). Araks valley: Fleischer (1900) (as *S.frontalis* v. *araxicola*); Heyden et al. (1906) (as S.frontalisa.araxicola Fleisch.); Winkler (1927) (as S.frontalisa.araxicola Fleisch.); Fürsch et al. (1967) (as *S.araxicola* Fleischer).

**Scymnus (Scymnus) frontalis (Fabricius, 1787)**

*Coccinellafrontalis* Fabricius, 1787

*Scymnusquadrivulneratus* Mulsant, 1850 (synonymized by Fürsch et al. (1967))

**Literature data.** Armenia: Schneider and Leder (1879) (Helenowka (=Sevan)); Jacobson (1915) (Yerevan); Kovář (2007). Caucasus: Heyden et al. (1891) (as *S.frontalisbimaculatus* Mot.); Iablokoff-Khnzorian (1983); Winkler (1927) (as *S. 4-vulneratus* Muls.).

**Scymnus (Scymnus) inderihensis Mulsant, 1850**

**Literature data.** Armenia: Iablokoff-Khnzorian (1983).

**Scymnus (Scymnus) interruptus (Goeze, 1777)**

*Coccinellainterrupta* Goeze, 1777

**Literature data.** Armenia: Iablokoff-Khnzorian (1983); Kovář (2007).

**Scymnus (Scymnus) magnomaculatus Fürsch, 1958**

*Scymnusquadriguttatus* Capra, 1924

**Literature data.** Armenia: Iablokoff-Khnzorian (1983) (as *S.quadriguttatus* Capra, 1924); Kovář (2007).

[**Scymnus (Scymnus) manipulus Fürsch & Kreissl, 1967**]

**Literature data.** Araks valley: Fürsch et al. (1967).

**Scymnus (Scymnus) pallipes Mulsant, 1850**

**Literature data.** Armenia: Fürsch et al. (1967) (Saderaki, Suhulta). Caucasus: Mulsant (1850); Heyden et al. (1891); Heyden et al. (1906) (as *S.frontalispallipes* Muls.); Winkler (1927) (as S.frontalisa.pallipes Muls.).

**Remark.**Fürsch et al. (1967) report that two paralectotypes of this species are from Saderaki and Suhulta in Armenia. The former name certainly refers to Sadarak in Azerbaijani exclave of Nakhchivan, while the location of the latter is unclear to us (Fürsch et al. (1967) also pointed out that the name of the locality on the label is difficult to decipher).

**Scymnus (Scymnus) rubromaculatus (Goeze, 1777)**

*Coccinellarubromaculata* Goeze, 1777

**Literature data.** Armenia: Schneider and Leder (1879) (Echmiadzin, Yerevan); Radde (1899) (Echmiadzin); Jacobson (1915) (Yerevan).

[**Scymnus (Scymnus) suffrianioides apetzoides Capra & Fürsch, 1967**]

*Scymnusapetzoides* Capra & Fürsch, 1967

**Literature data.** Caucasus: Iablokoff-Khnzorian (1983).

#### ﻿Tribe Platynaspini Mulsant, 1846

***Platynaspisluteorubra* (Goeze, 1777)**

*Coccinellaluteorubra* Goeze, 1777

*Scymnusspectabilis* Faldermann, 1837

**Literature data.** Armenia: Schneider and Leder (1879) (Yerevan); Weise (1885); Jacobson (1915) (Yerevan); Kovář (2007). Transcaucasia: Faldermann (1837) (as *Scymnusspectabilis* Fald.). Caucasus: Winkler (1927); Iablokoff-Khnzorian (1983).

#### ﻿Tribe Hyperaspidini Mulsant, 1846

[***Hyperaspiscampestris* (Herbst, 1783)**]

*Coccinellacampestris* Herbst, 1783

**Literature data.** Caucasus: Winkler (1927).

[***Hyperaspiscaucasica* Crotch, 1874**]

**Literature data.** Caucasus: Heyden et al. (1891) (as *Oxynychuserythrocephaluscaucasicus* Crotch).

[***Hyperaspiserythrocephala* (Fabricius, 1787)**]

*Coccinellaerythrocephala* Fabricius, 1787

**Literature data.** Caucasus: Heyden et al. (1906) (as *Oxynychuserythrocephalus* a. *Guillardi* Muls.); Winkler (1927) (as *Oxynychuserythrocephalus* F.).

***Hyperaspisfemorata* (Motschulsky, 1837)**

*Coccinellafemorata* Motschulsky, 1837

*Hyperaspisdesertorum* v. *collaris* Fleischer, 1900 (synonymized by Iablokoff-Khnzorian (1971))

*Hyperaspisinaudax* Mulsant, 1853 (synonymized by Iablokoff-Khnzorian (1971))

**Literature data.** Armenia: Weise (1885) (as *H.reppensis* v. *femorata* Motsch.); Iablokoff-Khnzorian (1971, 1983); Kovář (2007). Araks valley: Radde (1899) (as H.reppensisvar.femorata Mot.); Fleischer (1900) (as *H.desertorum* v. *collaris*); Heyden et al. (1906) (as Hyperaspisdesertoruma.collaris Fleisch.). Caucasus: Victor (1837); Mulsant (1850); Mulsant (1853) (as *H.inaudax*); Heyden et al. (1891, 1906) (as *H.reppensis* v. *femorata* Motsch. and *H.reppensisinaudax* Muls.).

***Hyperaspishisteroides* (Faldermann, 1837)**

*Scymnushisteroides* Faldermann, 1837

**Literature data.** Armenia: Iablokoff-Khnzorian (1971, 1983); Kovář (2007). Transcaucasia: Faldermann (1837) (as *Scymnushisteroides*).

[***Hyperaspispolita* Weise, 1885**]

**Literature data.** Caucasus: Heyden et al. (1906) (as *H.transversoguttata* v. *10-guttata* Fleischer); Winkler (1927) (as *H.transversoguttata* a. *10 guttata* Fleisch.); Iablokoff-Khnzorian (1971).

***Hyperaspistransversoguttata* Weise, 1878**

**Literature data**: Armenia: Iablokoff-Khnzorian (1971) (Meghri region); Kovář (2007). Lower Araks: Iablokoff-Khnzorian (1983). Caucasus: Weise (1879); Heyden et al. (1891, 1906).

#### ﻿Tribe Diomini Gordon, 1999

***Diomusrubidus* (Motschulsky, 1837)**

*Scymnusrubidus* Motschulsky, 1837

**Literature data.** Armenia: Kovář (2007). Eastern Caucasus: Iablokoff-Khnzorian (1983). Caucasus: Heyden et al. (1891, 1906); Winkler (1927).

#### ﻿Tribe Chilocorini Mulsant, 1846

***Chilocorusbipustulatus* (Linnaeus, 1758)**

*Coccinellabipustulata* Linnaeus, 1758

**Literature data.** Armenia: Schneider and Leder (1879) (Echmiadzin); Radde (1899) (Echmiadzin); Jacobson (1915) (Yerevan); Kovář (2007). Caucasus: Iablokoff-Khnzorian (1983).

[***Chilocorusrenipustulatus* (Scriba, 1791)**]

*Coccinellarenipustulata* Scriba, 1791

**Literature data.** Transcaucasia: Iablokoff-Khnzorian (1983).

***Exochomusoctosignatus* (Gebler, 1830)**

*Coccinellaoctosignata* Gebler, 1830

**Literature data.** Armenia: Weise (1885); Jacobson (1915) (Yerevan); Tobias (1975) (Yerevan, as a host of *D.coccinellae*); Iablokoff-Khnzorian (1983); Kovář (1995, 2007). Araks valley: Radde (1899). Caucasus: Heyden et al. (1906) (as *Brumus 8-signatus* a. *conjunctus* Fleisch.).

***Exochomusquadriguttatus* Fleischer, 1900**

**Literature data.** Armenia: Iablokoff-Khnzorian (1983) (as E.quadripustulatusssp.quadriguttatus); Kovář (1995, 2007). Araks valley: Fleischer (1900) (as *E. 4-pustulatus* v. *4-guttatus*).

***Exochomusquadripustulatus* (Linnaeus, 1758)**

*Coccinellaquadripustulata* Linnaeus, 1758

**Literature data.** Armenia: Jacobson (1915) (Yerevan); Kovář (1995, 2007). Transcaucasia: Iablokoff-Khnzorian (1983). Caucasus: Weise (1885) (as *Exochomusquadripustulatus* v. *ibericus* Motsch.); Heyden et al. (1891, 1906) (as *Exochomusquadripustulatus* v. *ibericus* Motsch.).

***Exochomusundulatus* Weise, 1878**

**Literature data.** Armenia: Iablokoff-Khnzorian (1983). Caucasus: Weise (1879, 1885); Heyden et al. (1891, 1906); Winkler (1927) (as *Anexochomusundulatus* Ws.).

***Parexochomusmelanocephalus* (Zubkov, 1833)**

*Coccinellamelanocephala* Zubkov, 1833

**Literature data.** Armenia: Iablokoff-Khnzorian (1983); Kovář (2007). Caucasus: Heyden et al. (1891).

***Parexochomusnigripennis* (Erichson, 1843)**

*Chilocorusnigripennis* Erichson, 1843

**Literature data.** Armenia: Jacobson (1915) (as Exochomusflavipessubsp.nigripennis Er.) (Yerevan); Iablokoff-Khnzorian (1983). Araks valley: Radde (1899) (as Exochomusflavipesvar.nigripennis Er.).

***Parexochomusnigromaculatus* (Goeze, 1777)**

*Coccinellanigromaculata* Goeze, 1777

*Exochomuscollaris* Küster, 1849

**Literature data.** Armenia: Schneider and Leder (1879) (Echmiadzin, Yerevan, Tarstschai); Radde (1899) (as *Exochomusflavipes* Thnb.) (Yerevan, Echmiadzin); Jacobson 1915) (as *Exochomusflavipes* Thunb.) (Yerevan); Kovář (2007). Caucasus: Heyden et al. (1891, 1906) (as *Exochomusflavipes* v. *collaris* Küst.); Iablokoff-Khnzorian (1983).

***Parexochomuspubescens* (Küster, 1848)**

*Exochomuspubescens* Küster, 1848

**Literature data.** Armenia: Jacobson (1915) (Yerevan). Araks valley: Radde (1899); Iablokoff-Khnzorian (1983).

#### ﻿Tribe Sticholotidini Weise, 1901

***Coelopterusarmeniacus* Weise, 1894**

**Literature data.** Armenia: Jacobson (1915) (Yerevan); Winkler (1927) (Yerevan); Iablokoff-Khnzorian (1983) (as a synonym of *C.salinus* Mulsant, 1853); Kovář (2007). Araks valley: Weise (1894); Heyden et al. (1906).

***Pharoscymnusarmenus* Iablokoff-Khnzorian, 1970**

**Literature data.** Armenia: Iablokoff-Khnzorian (1970b, 1983); Kovář (2007).

**Remarks.** Described based on specimens from the Kapan region (Syunik province) (holotype) and Yeghegnadzor region (Vayots Dzor province) (paratype) in Armenia.

#### ﻿Tribe Coccidulini Mulsant, 1846

***Coccidulalithophiloides* Reitter, 1890**

**Literature data.** Armenia: Jacobson (1915) (Yerevan); Iablokoff-Khnzorian (1983) (Araks valley from Yerevan to Meghri); Kovář (2007); Szawaryn et al. (2021) (Echmiadzin, Yerevan). Araks: Heyden et al. (1891, 1906); Winkler (1927).

***Coccidularufa* (Herbst, 1783)**

*Dermestesrufus* Herbst, 1783

*Coccidulaunicolor* Reitter, 1890

**Literature data.** Armenia: Jacobson (1915) (as *C.unicolor* Rt.) (Yerevan). Araks: Heyden et al. (1891) (as *C.rufa* v. *unicolor* Reitt.). Caucasus: Reitter (1890); Winkler (1927) (as *C.unicolor* Rtt.).

***Coccidulascutellata* (Herbst, 1783)**

*Chrysomelascutellata* Herbst, 1783

**Literature data.** Armenia: Szawaryn et al. (2021) (Yerevan).

#### ﻿Tribe Tetrabrachini Kapur, 1948

***Tetrabrachysaraxis* (Reitter, 1897)**

*Lithophilusaraxis* Reitter, 1897

**Literature data.** Armenia: Jacobson (1915) (Yerevan); Iablokoff-Khnzorian (1983) (Hrazdan valley, Mt. Aragats slopes, lake Sevan shores); Kovář (2007). Araks valley: Radde (1899); Heyden et al. (1906); Winkler (1927).

***Tetrabrachysbipustulatus* (Barovskij, 1909)**

*Lithophilusbipustulatus* Barovskij, 1909

**Literature data.** Armenia: Iablokoff-Khnzorian (1974, 1983) (surroundings of Yerevan); Kovář (2007).

**Remarks.** According to Iablokoff-Khnzorian (1974), a distinct subspecies *Lithophilus* (=*Tetrabrachys*) *bipustulatus armeniacus* occurs in Armenia.

[***Tetrabrachyscaucasicus* (Weise, 1878)**]

*Lithophiluscaucasicus* Weise, 1878

**Literature data.** Caucasus: Heyden et al. (1906); Winkler (1927).

***Tetrabrachysconnatus* (Creutzer, 1796)**

*Tritomaconnata* Creutzer, 1796

**Literature data.** Armenia: Radde (1899) (Darachichag (=Tsaghkadzor)); Jacobson (1915) (Yerevan).

**Remark.** In Jacobson’s (1915) list of the distribution records of *T.connatus*, Yerevan is preceded by a question mark.

***Tetrabrachysmajor* (Crotch, 1874)**

*Lithophilusmajor* Crotch, 1874

**Literature data.** Armenia: Jacobson (1915) (Yerevan). Araks valley: Heyden et al. (1906).

[***Tetrabrachysweisei* (Reitter, 1880)**]

*Lithophilusweisei* Reitter, 1880

**Caucasus literature data.**Heyden et al. (1906); Winkler (1927).

### ﻿Comparison of Coccinellidae faunas of Armenia, Azerbaijan, and Georgia

The checklist presented above contains 84 species that have been reported from Armenia and 14 additional species with imprecise locations (Transcaucasia, Araks valley, the Caucasus) indicating that they may or may not include Armenia. Four species reported from Armenia or adjacent areas by Jacobson (1915) and two reported by Kovář (2007) are not included, listed in Table 1 with the reasons for their exclusion.

**Table 1.** Species reported from Armenia or adjacent regions, but not included in the present checklist.

Species	Justification for exclusion
*Oenopiadoublieri* (Mulsant, 1846)	Jacobson (1915) reported a doubtful (with a question mark) record of *O.doublieri* from Yerevan, citing Schneider and Leder (1879) as a source of this information. Indeed, these authors reported *O.doublieri*, however, not from the Yerevan area, but from the North Caucasus (Karasu village in Kabardino-Balkaria (Russia)).
*Scymniscusarmeniacus* (Canepari, 1979)	Kovář (2007) reported this species as occurring in Armenia, probably due to its specific epithet. However, Canepari (1979) described *S.armeniacus* (as Nephus (Sidis) armeniacus) based on a single male specimen collected in Elisabetspol (today’s Ganja in Azerbaijan). Canepari derived the name *armeniacus* from the ancient region of Armenia that covered much more extensive area than present-day Armenia.
Scymnus (Scymnus) rufipes (Fabricius, 1798)	Jacobson (1915) reported this species from Transcaucasia. Its identity is uncertain given some of its synonyms listed by Jacobson (*S.corpulentus* Muls., *S.suffrianioides* J. Sahlb.). According to Kovář (2007), *S.corpulentus* is considered a synonym of *S.apetzi* Mulsant and *S.suffrianioides* is a valid species different from *S.rufipes*.
*Hyperaspisdesertorum* Weise, 1885	Jacobson (1915) reported *H.desertorum* from Yerevan, which may refer either to this species or, more likely, to *H.femorata* Motschulsky, as indicated by one of the synonyms mentioned (H.desertorumab.collaris Fleisch.).
*Hyperaspisreppensis* (Herbst, 1783)	Jacobson’s (1915) report of *H.reppensis* from Yerevan cannot be assigned to this or other related species due to the long list of synonyms given, which are currently recognized as several species (e.g., *H.stigma* A. Ol., *H.pseudopustulata* Muls., *H.hoffmannseggi* Grav., *H.histeroides* Fald., *H.illecebrosa* Chevr., *H.femorata* Motsch., *H.quadrimaculata* Redt.).
*Pharoscymnuskoenigi* Iablokoff-Khnzorian, 1970	According to Kovář’s (2007) catalogue, *P.koenigi* occurs in both the Asiatic part of Turkey and Armenia. However, the holotype and three paratypes of *P.koenigi*, all collected in Oltu (eastern Turkey) (Iablokoff-Khnzorian 1970c), are probably the only known specimens of this species.

A comparison of the reported ladybird fauna of Armenia with that of other Transcaucasian states (Azerbaijan and Georgia) is shown in Table 2. The total number of species for all the Transcaucasian countries is 116, with 84, 92, and 90 species reported from Armenia, Azerbaijan, and Georgia, respectively. Thus, the recognized ladybird fauna of Armenia is somewhat poorer than that of Azerbaijan and Georgia. However, it should be borne in mind that Armenia occupies a noticeably smaller area than the other two states: Georgia is more than twice and Azerbaijan almost three times the size of Armenia.

Approximately 12% of ladybird species reported from Transcaucasia (14 of 116 species) can be considered endemic or near-endemic to the Caucasus ecoregion (Table 3). Ten of them have been reported from Armenia (11.9% of the 84 species reported), four from Azerbaijan (4.3% of the 92 species reported), and six from Georgia (6.7% of the 90 species reported).

Another group of special interest are ladybird species non-native to the region. To our knowledge, eight such species have been reported from Transcaucasia, but the occurrence of two of them there seems unlikely, so they are not included in the list of the Transcaucasian Coccinellidae in Table 2. One of these, *Chilocorussimilis* (Rossi, 1790), was reported by Schneider and Leder (1879) from Lailashi, Georgia, most probably as a result of a misidentification of *C.renipustulatus* (Scriba, 1791). The second species, *Henosepilachnavigintioctopunctata* (Fabricius, 1775), a herbivorous ladybird widely distributed in the eastern part of the Palaearctic and in the Oriental and Australian regions (Kovář 2007), was reported from Azerbaijan in an unpublished thesis cited by Snegovaya and Zare Khormizi (2022). This report likely pertains to a different species of Epilachnini. The presence in the region of the remaining six species is likely, given that each of them has been introduced in the Caucasus in the past (Table 4). All six species have been reported from Georgia, two (*Harmoniaaxyridis* and *Rhyzobiuslophanthae*) from Azerbaijan, and only one (*H.axyridis*) from Armenia.

To conclude, the recognized ladybird fauna of Armenia, although slightly less abundant in species than those of Azerbaijan and Georgia, appears to be diverse, with a high proportion of endemic species. On the other hand, only one alien ladybird species, the harlequin ladybird (*H.axyridis*), has so far been reported from this country. The field survey revealed that this highly invasive species has become common and widespread in many parts of Armenia. The survey also shows the presence in Armenia of two ladybird species, *Anatisocellata* and *Tytthaspissedecimpunctata*, which had not previously been reported from the region. Further field research and examination of existing insect collections would certainly increase the number of Armenian ladybird species.

**Table 2.** Coccinellidae reported from the Transcaucasian countries. The Armenian data are taken from the present checklist, while those for Azerbaijan and Georgia are primarily based on recent checklists by Snegovaya and Zare Khormizi (2022) and Migeon and Arabuli (2022), respectively. A few reports from other sources are marked and footnoted. [**A**] after the species name indicates a species alien to the region, [**E**] indicates presumed endemic or nearly endemic species. Asterisks (*) denote new country records. The footnotes are explained at the end of the table.

Species	Armenia	Azerbaijan	Georgia
**Microweiseinae**			
**Serangiini**			
*Serangiummontazerii* Fürsch, 1995 [**A**]			+
**Coccinellinae**			
**Stethorini**			
*Stethorusgilvifrons* (Mulsant, 1850)	+	+	+
*Stethoruspusillus* (Herbst, 1797)	+	+	+
**Coccinellini**			
*Adaliabipunctata* (Linnaeus, 1758)	+	+	+
*Adaliadecempunctata* (Linnaeus, 1758)	+	+	+
*Anatisocellata* (Linnaeus, 1758)*	+	+	+
*Anisostictacaucasica* (Fleischer, 1900) [**E**]	+		
*Anisostictanovemdecimpunctata* (Linnaeus, 1758)	+	+	+
*Aphidectaobliterata* (Linnaeus, 1758)	+	+	+
*Bulaealichatschovii* (Hummel, 1827)	+	+	+
*Calviadecemguttata* (Linnaeus, 1767)	+	+	+
*Calviaquatuordecimguttata* (Linnaeus, 1758)	+	+	+
*Calviaquindecimguttata* (Fabricius, 1777)		+	+
*Ceratomegillaapicalis* (Weise, 1879)	+	+	+
*Ceratomegillanotata* (Laicharting, 1781)	+	+	+
*Ceratomegillaschelkovnikovi* (Dobzhansky, 1927) [**E**]	+		+^1^
*Ceratomegillaschneideri* (Weise, 1878) [**E**]	+	+	+
*Ceratomegillaundecimnotata* (Schneider, 1792)	+	+	+
*Coccinellaalpigrada* (Iablokoff-Khnzorian, 1957) [**E**]	+		
*Coccinellahieroglyphica* Linnaeus, 1758		+	+
*Coccinellamagnifica* Redtenbacher, 1843	+	+^2^	+
*Coccinellaquinquepunctata* Linnaeus, 1758	+	+	+
*Coccinellasaucerottii* Mulsant, 1850		+	
*Coccinellaseptempunctata* Linnaeus, 1758	+	+	+
*Coccinellaundecimpunctata* Linnaeus, 1758	+	+	
*Coccinulaquatuordecimpustulata* (Linnaeus, 1758)	+	+	+
*Coccinulasinuatomarginata* (Faldermann, 1837)	+	+	+
*Halyziasedecimguttata* (Linnaeus, 1758)	+	+	+
*Harmoniaaxyridis* (Pallas, 1773) [**A**]	+	+	+
*Harmoniaconformis* (Boisduval, 1835) [**A**]			+
*Harmoniaquadripunctata* (Pontoppidan, 1763)		+	+
*Hippodamiaseptemmaculata* (DeGeer, 1775)		+	
*Hippodamiatredecimpunctata* (Linnaeus, 1758)	+	+	+
*Hippodamiavariegata* (Goeze, 1777)	+	+	+
*Myrrhaoctodecimguttata* (Linnaeus, 1758)	+	+	+
*Myziaoblongoguttata* (Linnaeus, 1758)	+	+	+
*Oenopiabissexnotata* (Mulsant, 1850)		+	
*Oenopiaconglobata* (Linnaeus, 1758)	+	+	+
*Oenopiaimpustulata* (Linnaeus, 1758)	+	+	+
*Oenopialynceaagnatha* (Rosenhauer, 1808)	+	+	+
*Oenopiaoncina* (Olivier, 1808)	+	+	+
*Propyleaquatuordecimpunctata* (Linnaeus, 1758)	+	+	+
*Psylloboravigintiduopunctata* (Linnaeus, 1758)	+	+	+
*Sospitavigintiguttata* (Linnaeus, 1758)			+
*Tytthaspissedecimpunctata* (Linnaeus, 1761)*	+	+	+
*Vibidiaduodecimguttata* (Poda von Neuhaus, 1761)	+	+	+
**Epilachnini**			
*Chnootribaelaterii* (Rossi, 1794)		+	+
*Cynegetisimpunctata* (Linnaeus, 1767)		+	
*Henosepilachnaargus* (Geoffroy, 1785)	+	+	+
*Subcoccinellavigintiquatuorpunctata* (Linnaeus, 1758)	+	+	+
**Noviini**			
*Noviuscardinalis* (Mulsant, 1850) [**A**]			+
**Scymnini**			
*Clitostethusarcuatus* (Rossi, 1794)	+	+	+
Nephus (Bipunctatus) bipunctatus (Kugelann, 1794)		+	+
Nephus (Geminosipho) reunioni (Fürsch, 1974) [**A**]		+	+^3^
Nephus (Nephus) ludyi (Weise, 1879)	+		
Nephus (Nephus) quadrimaculatus (Herbst, 1783)	+	+	+
Nephus (Nephus) redtenbacheri (Mulsant, 1846)		+	+
Nephus (Sidis) caucasicus (Weise, 1929) [**E**]	+		
*Scymniscusarmeniacus* (Canepari, 1979) [**E**]		+^4^	
*Scymniscusbiflammulatus* (Motschulsky, 1837)	+	+	+
*Scymniscusbiguttatus* (Mulsant, 1850)	+	+	+
Scymnus (Mimopullus) pharaonis Motschulsky, 1851	+		
Scymnus (Neopullus) haemorrhoidalis Herbst, 1797	+	+	+
Scymnus (Neopullus) limbatus Stephens, 1832	+	+	+
Scymnus (Neopullus) testaceus Motschulsky, 1837	+	+	+^5^
Scymnus (Pullus) argutus Mulsant, 1850	+	+	+
Scymnus (Pullus) auritus Thunberg, 1795		+	+
Scymnus (Pullus) ferrugatus (Moll, 1785)			+
Scymnus (Pullus) fraxini Mulsant, 1850	+	+	+
Scymnus (Pullus) subvillosus (Goeze, 1777)	+	+	+
Scymnus (Pullus) suturalis Thunberg, 1795	+	+	+^5^
Scymnus (Scymnus) apetzi Mulsant, 1846	+	+	+
Scymnus (Scymnus) femoralis (Gyllenhal, 1827)		+	
Scymnus (Scymnus) flavicollis Redtenbacher, 1843	+		
Scymnus (Scymnus) frontalis (Fabricius, 1787)	+	+	+
Scymnus (Scymnus) inderihensis Mulsant, 1850	+		
Scymnus (Scymnus) interruptus (Goeze, 1777)	+	+	+
Scymnus (Scymnus) magnomaculatus Fürsch, 1958	+	+	+
Scymnus (Scymnus) marginalis (Rossi, 1794)			+
Scymnus (Scymnus) nigrinus Kugelann, 1794		+	+
Scymnus (Scymnus) pallipes Mulsant, 1850	+		
Scymnus (Scymnus) rubromaculatus (Goeze, 1777)	+	+	+
Scymnus (Scymnus) rufipes (Fabricius, 1798)		+	
**Platynaspini**			
*Platynaspisluteorubra* (Goeze, 1777)	+	+	+
**Hyperaspidini**			
*Hyperaspiscampestris* (Herbst, 1783)			+
*Hyperaspiscaucasica* Crotch, 1874 [**E**]		+^6^	
*Hyperaspiserythrocephala* (Fabricius, 1787)		+	+
*Hyperaspisfemorata* (Motschulsky, 1837)	+	+	+
*Hyperaspishisteroides* (Faldermann, 1837)	+	+	
*Hyperaspisreppensis* (Herbst, 1783)		+	+
*Hyperaspistransversoguttata* Weise, 1878	+	+	+
**Diomini**			
*Diomusrubidus* (Motschulsky, 1837)	+	+	+
**Chilocorini**			
*Chilocorusbipustulatus* (Linnaeus, 1758)	+	+	+
*Chilocorusrenipustulatus* (Scriba, 1791)		+	+
*Exochomusoctosignatus* (Gebler, 1830)	+	+	+
*Exochomusquadriguttatus* Fleischer, 1900 [**E**]	+		+^7^
*Exochomusquadripustulatus* (Linnaeus, 1758)	+	+	+
*Exochomusundulatus* Weise, 1878	+	+	+
*Parexochomusmelanocephalus* (Zubkov, 1833)	+	+	+^8^
*Parexochomusnigripennis* (Erichson, 1843)	+		
*Parexochomusnigromaculatus* (Goeze, 1777)	+	+	+
*Parexochomuspubescens* (Küster, 1848)	+	+	
**Sticholotidini**			
*Coelopterusarmeniacus* Weise, 1894 [**E**]	+		
*Pharoscymnusarmenus* Iablokoff-Khnzorian, 1970 [**E**]	+		+
*Pharoscymnussmirnovi* Dobzhansky, 1927		+	+
**Coccidulini**			
*Coccidulalithophiloides* Reitter, 1890 [**E**]	+	+	
*Coccidularufa* (Herbst, 1783)	+	+	+
*Coccidulascutellata* (Herbst, 1783)	+	+	+
*Rhyzobiuslophanthae* (Blaisdell, 1892) [**A**]		+	+
**Tetrabrachini**			
*Tetrabrachysaraxis* (Reitter, 1897) [**E**]	+	+	
*Tetrabrachysbipustulatus* (Barovskij, 1909)	+		
*Tetrabrachyscaucasicus* (Weise, 1878) [**E**]			+
*Tetrabrachyscoloratus* Fürsch, 1960		+	
*Tetrabrachysconnatus* (Creutzer, 1796)	+	+	+
*Tetrabrachysmajor* (Crotch, 1874)	+		
*Tetrabrachysweisei* (Reitter, 1880) [**E**]			+
No. species: 116	84	92	90

^1^ Reported by Dobzhansky (1927) from Borjomi and Bakuriani in Georgia and Mamison Pass on the Georgian-Russian border and by Kovář (2007) generally from Georgia. ^2^ Reported by Schneider and Leder (1879) from Baku district, Azerbaijan. ^3^ Reported from Georgia by Kovář (2007). ^4^ Reported by Canepari (1979) from Elisabetspol (now Ganja), Azerbaijan. ^5^ From Georgia reported by Schneider and Leder (1879) and Merkviladze and Kvavadze (2002). ^6^ Reported from Nukha (now Shaki), Azerbaijan by Motschulsky (Victor 1837) (as *Coccinella 6 pustulata* Victor). Kovář’s (2007) report from Georgia was not included, as it probably follows Motschulsky’s (Victor 1837) description of the site as ‘Noucha en Géorgie’. ^7^ Reported by Kovář (1995) from Gagra in Abkhazia (formally part of Georgia) and by Iablokoff-Khnzorian (1983) and Merkviladze and Kvavadze (2002) (as E.quadripustulatusssp.quadriguttatus Fleisch, 1900) from several regions of Georgia. ^8^ Reported by Merkviladze and Kvavadze (2002) from several regions in Georgia.

**Table 3.** Coccinellidae species with a known range restricted to or only slightly exceeding the Caucasus ecoregion.

Species	Remarks on distribution and nomenclature
*Anisostictacaucasica* (Fleischer, 1900)	For a long time, this ladybird was known only from a single type specimen that, according to Iablokoff-Khnzorian (1982), was collected by Fleischer in the Armenian part of the Araks valley. Further specimens were reported by Fürsch (1977) from Dizin in northern Iran.
*Ceratomegillaschelkovnikovi* (Dobzhansky, 1927)	It seems that the specimens collected by Dobzhansky (1927) in several locations in Armenia, Georgia, and the Russian part of the Caucasus (upper course of the Belaya River) are the only known specimens of this species.
*Ceratomegillaschneideri* (Weise, 1878)	This species is probably a Caucasian endemic. Apart from Armenia, Schneider and Leder (1879) reported it from Georgia and Azerbaijan and Iablokoff-Khnzorian (1983) added the Russian territories of Kabardino-Balkaria, Kuban region and Ossetia in the North Caucasus. Bieńkowski (2018: fig. 11C) presented a photograph of a specimen from the Republic of Adygea (NW of the North Caucasus, Russia).
*Coccinellaalpigrada* (Iablokoff-Khnzorian, 1957)	Khnzorian (1957) reported several Armenian sites for *C.alpigrada*. For a long time, these were the only known sites of this species until Kovář (2005) reported it from the Erzurum Province of Turkey (Armenian Upland).
*Nephuscaucasicus* (Weise, 1929)	This species was described (as *Scymnusplagiatus* Weise in Schneider & Leder, 1879) based on specimens collected near Karasu village (Kabardino-Balkaria, the North Caucasus, Russia) and Yerevan (Armenia). More recently, it was also reported from Tehran Province in northern Iran (Jafari et al. 2013).
*Scymniscusarmeniacus* (Canepari, 1979)	It seems that the type specimen from Ganja in Azerbaijan (Canepari 1979) is the only known specimen of this species.
*Hyperaspiscaucasica* Crotch, 1874	Motschulsky (Victor 1837) found this species in the environs of present-day Shaka in Azerbaijan and described it as *Coccinella 6 pustulata*. Subsequently, due to homonymy, Crotch (1874) replaced this name with *H.caucasica*. Kovář (2007) considered *Hyperaspisassimilis* Zaslavskij, 1966 from Tajikistan as a synonym of *H.caucasica*, while Iablokoff-Khnzorian (1983) treated both *H.caucasica* and *H.assimilis* as synonyms of *H.guttulata* Fairmaire, 1870, a species reported from the western Mediterranean region, Tajikistan, and Mongolia (Kovář 2007). A comparative examination of *H.caucasica*, *H.assimilis*, and *H.guttulata* would need to be carried out to establish their identities.
*Exochomusquadriguttatus* Fleischer, 1900	*Exochomusquadriguttatus* appears to be endemic to the Caucasus ecoregion or its range extends slightly beyond this area. It was reported from the western North Caucasus, western Georgia, Armenia, and north-eastern Anatolia (Iablokoff-Khnzorian 1983; Kovář 1995; Merkviladze and Kvavadze 2002).
*Coelopterusarmeniacus* Weise, 1894	*Coelopterusarmeniacus* was described based on two specimens collected in the Araks valley. Later authors consistently reported it from Armenia but, surprisingly, Kovář (2007) also mentioned Israel.
*Pharoscymnusarmenus* Iablokoff-Khnzorian, 1970	It is known from Armenia and eastern Georgia (Iablokoff-Khnzorian 1970b, 1983).
*Coccidulalithophiloides* Reitter, 1890	*Coccidulalithophiloides* was described from Ordubad (Azerbaijan exclave of Nakhchivan). According to Iablokoff-Khnzorian (1983), it is common in Armenia along the Araks valley. It was also reported from several provinces of Iran (Kermanshah, Lorestan, Isfahan, and Fars; Biranvand et al. 2024), where it was described by Duverger (1983) as *Lithophilusnaviauxi*.
*Tetrabrachysaraxis* (Reitter, 1897)	Iablokoff-Khnzorian (1983) stated that *T.araxis* was described from Armenia, but Reitter’s (1897) description was based on specimens collected in the Araks valley near Ordubad, i.e., in the Azerbaijan exclave of Nakhchivan. Nonetheless, the former author mentioned several other locations of this species within the current borders of Armenia. *Tetrabrachysanatolicus* (Pic, 1901), considered a synonym of *T.araxis* both by Iablokoff-Khnzorian (1983) and Kovář (2007), was described from Konya in southern Turkey, more than 1,000 km W-WS of the Transcaucasian locations. The identity of *T.anatolicus* needs to be examined.
*Tetrabrachyscaucasicus* (Weise, 1878)	Description of this species was based on a single specimen collected in Borjomi in Georgia (Schneider and Leder 1879). Merkviladze and Kvavadze (2002) reported it also from the region of Tbilisi, and Kovář (2007) mentioned it generally from the Asiatic part of Turkey.
*Tetrabrachysweisei* (Reitter, 1880)	Reitter (1880) mentioned in general that the specimen he used to describe *T.weisei* was collected by Leder in the Caucasus. Merkviladze and Kvavadze (2002) reported it from the Tbilisi district of Georgia, and Iablokoff-Khnzorian (1974, 1983) also from Crimea.

**Table 4.** Non-native Coccinellidae reported from Transcaucasia.

Species	Information on introductions in the region
*Serangiummontazerii* Fürsch, 1995	Widely released in the Black Sea coast of the Caucasus after 1973 (as *S.parcesetosum* (Sicard, 1929)) (Booth and Polaszek 1996; Bieńkowski and Orlova-Bienkowskaja 2020). Its presence in western Georgia was confirmed by Migeon and Arabuli (2022).
*Harmoniaaxyridis* (Pallas, 1773)	Released after 1927 in Georgia and then at the Black Sea coast of Russia (Bieńkowski and Orlova-Bienkowskaja 2020). Its presence in eastern Georgia was confirmed by Merkviladze and Kvavadze (2002). The European invasive population has been spreading in the Caucasus since ~ 2012 (Belyakova and Reznik 2013; Ukrainsky 2013).
*Harmoniaconformis* (Boisduval, 1835)	Released in Georgia after 1958 (Bieńkowski and Orlova-Bienkowskaja 2020). Its presence in western Georgia was confirmed by Merkviladze and Kvavadze (2002).
*Noviuscardinalis* (Mulsant, 1850)	Released in the Caucasus after 1931 (Bieńkowski and Orlova-Bienkowskaja 2020). Its presence in western Georgia was confirmed by Merkviladze and Kvavadze (2002).
*Nephusreunioni* (Fürsch, 1974)	Released in Georgia before 1987 (Bieńkowski and Orlova-Bienkowskaja 2020).
*Rhyzobiuslophanthae* (Blaisdell, 1892)	Released in the Caucasus after 1947 (Bieńkowski and Orlova-Bienkowskaja 2020). Its presence in western Georgia was confirmed by Merkviladze and Kvavadze (2002).

## ﻿Additional information

### Author ORCIDs

Shoghik Ghazaryan https://orcid.org/0009-0007-9071-6545

Marine Arakelyan https://orcid.org/0000-0002-6334-5714

Astghik Ghazaryan https://orcid.org/0009-0005-2840-0019

Jerzy Romanowski https://orcid.org/0000-0003-1050-6403

Krzysztof Turlejski https://orcid.org/0000-0001-6708-7815

Piotr Ceryngier https://orcid.org/0000-0003-1085-9751

### Data availability

All of the data that support the findings of this study are available in the main text.

## References

[B1] Belyakova NA Reznik SYa (2013) First record of the harlequin ladybird, *Harmoniaaxyridis* (Coleoptera: Coccinellidae) in the Caucasus. European Journal of Entomology 110: 699–702. 10.14411/eje.2013.093

[B2] Bieńkowski AO (2018) Key for identification of the ladybirds (Coleoptera: Coccinellidae) of European Russia and the Russian Caucasus (native and alien species). Zootaxa 4472(2): 233–260. 10.11646/zootaxa.4472.2.2 30313367

[B3] Bieńkowski AO Orlova-Bienkowskaja MJ (2020) History of the biodiversity of ladybirds (Coccinellidae) at the Black Sea coast of the Russian Caucasus in the last 120 years – does the landscape transformation and establishment of *Harmoniaaxyridis* have an impact? Insects 11: 824. 10.3390/insects11110824 PMC7700593 33238578

[B4] Biranvand A Fekrat L Vĕtrovec J Ghobari H Hamidi E Zare Khormizi M Azadbakht N Nedvĕd O Romasi F Ceryngier P (2024) Checklist and distribution of ladybirds (Coleoptera, Coccinellidae) in Iranian provinces. Zootaxa 5493(2): 101–128. 10.11646/zootaxa.5493.2.1 39646584

[B5] Booth RG Polaszek A (1996) The identities of ladybird beetle predators used for whitefly control, with note on some whitefly parasitoides, in Europe. In: Brighton Crop Protection Conference – Pests & Diseases – 1996, Volume I, Brighton (England), 18–21 November 1996, 69–74.

[B6] Canepari C (1979) Due nuove species di Scymnini palearctici: Nephus (Sidis) armeniacus e Scymnus (Pullus) folchinii (Coleoptera: Coccinellidae). Doriana 5(232): 1–5.

[B7] Ceryngier P Franz KW Romanowski J (2023) Distribution, host range and host preferences of *Dinocampuscoccinellae* (Hymenoptera: Braconidae): A worldwide database. European Journal of Entomology 120: 26–34. 10.14411/eje.2023.004

[B8] Che L Zhang P Deng S Escalona HE Wang X Li Y Pang H Vandenberg N Ślipiński A Tomaszewska W Liang D (2021) New insights into the phylogeny and evolution of lady beetles (Coleoptera: Coccinellidae) by extensive sampling of genes and species. Molecular Phylogenetics and Evolution 156: 107045. 10.1016/j.ympev.2020.107045 33352317

[B9] Crotch GR (1874) A Revision of the Coleopterous Family Coccinellidae. E. W. Janson, London, xv + 311 pp. 10.5962/bhl.title.8975

[B10] Dobzhansky Th (1927) Neue und wenig bekannte Coccinelliden. Revue Russe d’Entomologie 21: 212–217.

[B11] Duverger C (1983) Contribution a la connaissance des Coccinellidae d’Iran. Nouvelle Revue d’Entomologie 13: 73–93.

[B12] Faldermann F (1835) Additamenta entomologica ad faunam rossicam in itineribus jussu imperatoris augustissimi annis 1827–1831 a Cl. Ménétriés et Szovitz susceptis collecta, in lucem edita. Noveaux Mémoires de la Société Impériale des Naturalistes de Moscou 4: 1–310.

[B13] Faldermann F (1837) Fauna Entomologica Trans-Caucasica. Pars II. Coleoptera Trans-Caucasica. II. Heteromera. Noveaux Mémoires de la Société Impériale des Naturalistes de Moscou 5: 1–412.

[B14] Faldermann F (1838) Fauna Entomologica Trans-Caucasica. Coleoptera. Pars III. Auguste Semen, Moscou, 306 pp.

[B15] Fleischer A (1900) Neue Coccinelliden aus der Sammlung des kais. Rathes Herrn Edmund Reitter. Wiener entomologische Zeitung 19: 116–120. 10.5962/bhl.part.3442

[B16] Fürsch H (1977) Coccinellidenausbeuten aus Libanon und dem Iran im Museum Genf mit Beschreibung neuer Scymnini-Arten (Col. Cocc.). Revue Suisse de Zoologie 84: 645–657. 10.5962/bhl.part.91413

[B17] Fürsch H Kreissl E Capra F (1967) Revision einiger europäischer Scymnus (s.str.)-Arten. Mitteilungen der Abteilung für Zoologie und Botanik am Landesmuseum “Joanneum” in Graz 28: 207–259.

[B18] Hewitt GM (1999) Post-glacial re-colonization of European biota. Biological Journal of the Linnean Society 68: 87–112. 10.1111/j.1095-8312.1999.tb01160.x

[B19] Heyden L v Reitter E Weise J (1891) Catalogus Coleopterorum Europae, Caucasi et Armeniae rossicae. Edmund Reitter, Berlin-Mödling-Caen, viii + 812 pp.

[B20] Heyden Lv Reitter E Weise J (1906) Catalogus Coleopterorum Europae, Caucasi et Armeniae rossicae, edition secunda. Edmund Reitter, Berlin-Paskau-Caen, 775 pp.

[B21] Iablokoff-Khnzorian SM (1957) New species of beetles from the Armenian SSR and Nakh. ASSR. Academy of Sciences of Armenian SSR, Zoological Institute, Materials on the Study of Fauna of the Armenian SSR 3 (Zoological Collection 10): 153–183. [in Russian, with Armenian summary]

[B22] Iablokoff-Khnzorian SM (1969) Two new species of ladybird beetles from the Caucasus. Doklady Akademii Nauk Armyanskoi SSR 48: 247–250. [in Russian, with Armenian summary]

[B23] Iablokoff-Khnzorian SM (1970a) Two new species of the genus *Nephus* Muls. from USSR. Doklady Akademii Nauk Armyanskoi SSR 50: 118–121. [in Russian, with Armenian summary]

[B24] Iablokoff-Khnzorian SM (1970b) New species of beetles from Armenia and other lands of USSR. Academy of Sciences of Armenian SSR, Zoological Institute, Zoological Papers 15: 50–80. [in Russian, with Armenian and English summary]

[B25] Iablokoff-Khnzorian SM (1970c) Two new ladybird species from the Palaearctic. Doklady Akademii Nauk Armyanskoi SSR 50: 252–256. [in Russian, with Armenian summary]

[B26] Iablokoff-Khnzorian SM (1971) Synopsis des *Hyperaspis* Paléarctiques (Col. Coccinellidae). Annales de la Société entomologique de France (N.S.) 7: 163–200. 10.1080/21686351.1971.12278033

[B27] Iablokoff-Khnzorian SM (1972) Les types de Coccinellidae de la collection Motschulsky (Coléoptères Coccinellidae). Nouvelle Revue d’Entomologie 2: 163–184. 10.1080/21686351.1971.12278033

[B28] Iablokoff-Khnzorian SM (1974) Monographie der Gattung *Lithophilus* Froelich (Col. Coccinellidae). Entomologische Arbeiten aus dem Museum G. Frey Tutzing bei München 25: 148–243.

[B29] Iablokoff-Khnzorian SM (1980) Tribe Epilachnini (Coleoptera, Coccinellidae) in the fauna of the USSR. I. Entomologicheskoye Obozreniye 59: 297–310. [in Russian]

[B30] Iablokoff-Khnzorian SM (1981) Tribe Epilachnini (Coleoptera, Coccinellidae) in the fauna of the USSR. II. Entomologicheskoye Obozreniye 60: 849–859. [in Russian]

[B31] Iablokoff-Khnzorian SM (1982) Les Coccinelles Coléoptères – Coccinellidae Tribu Coccinellini des regions Palearctique et Orientale. Paris, Boubée, 568 pp.

[B32] Iablokoff-Khnzorian SM (1983) Review of the coccinellid fauna of the USSR. Academy of Sciences of Armenian SSR, Institute of Zoology, Zoological Papers 19: 94–161. [in Russian, with Armenian and English summary]

[B33] Jacobson GG (1915) Beetles of Russia and western Europe – a guide to beetle identification. Izd. A.F. Devrien, St. Petersburg, issue XI, 865–1024. [in Russian]

[B34] Jafari R Fursch H Zarei Khormizi M (2013) A checklist of the Scymninae (Coleoptera: Coccinellidae) of Iran. International Research Journal of Applied and Basic Sciences 5: 154–160.

[B35] Kalashian MYu Ghrejyan TL Karagyan GH (2017) Harlequin ladybird *Harmoniaaxyridis* Pall. (Coleoptera, Coccinellidae) in Armenia. Russian Journal of Biological Invasions 8: 313–315. 10.1134/S207511171704004X

[B36] Kalashian MYu Ghrejyan TL Karagyan GH (2019) Expansion of harlequin ladybird *Harmoniaaxyridis* Pall. (Coleoptera, Coccinellidae) in Armenia. Russian Journal of Biological Invasions 10: 153–156. 10.1134/S2075111719020073

[B37] Kalashian M Aghababyan K Zarikian N Gabrielyan B Arakelyan M Ghazaryan A (2023) Fauna of Armenia. In: Fayvush G (Ed.) Biodiversity of Armenia.Springer Nature Switzerland AG, 165–282. 10.1007/978-3-031-34332-2_5

[B38] Khnzorian SM (1957) New species of beetles from the Armenian SSR and Nakh. ASSR. Academy of Sciences of the Armenian SSR – Institute of Zoology, Materials for the Study of the Fauna of the Armenian SSR, III, Zoological Collection 10: 153–183. [in Russian, with Armenian summary]

[B39] Kovář I (1995) Revision of the genera *Brumus* Muls. and *Exochomus* Redtb. (Coleoptera, Coccinellidae) of the Palaearctic region. Part I. Acta Entomologica Musei Nationalis Pragae 44: 5–124.

[B40] Kovář I (2005) Revision of the Palaearctic species of the *Coccinellatransversoguttata* species group with notes on some other species of the genus (Coleoptera: Coccinellidae). Acta Entomologica Musei Nationalis Pragae 45: 129–164.

[B41] Kovář I (2007) Family Coccinellidae Latreille, 1807. In: Löbl I Smetana A (Eds) Catalogue of Palaearctic Coleoptera.Vol. 4. Elateroidea, Derodontoidea, Bostrichoidea, Lymexyloidea, Cleroidea, Cucujoidea. Apollo Books, Stentrup, Denmark, 71–74, 568–631.

[B42] Leder H (1879) Beitrag zur kaukasischen Käfer-Fauna. Verhandlungen der kaiserlich-königlichen zoologisch-botanischen Gesellschaft in Wien 29: 451–488.

[B43] Ménétries E (1832) Catalogue raisonné des objets de zoologie recueillis dans un voyage au Caucase et jusqu’aux frontières actuelles de la Perse entrepris par ordre de S. M. l’Empereur. Académie Impériale des Sciences, St.-Pétersbourg, 271 + xxxiii + iv pp. 10.5962/bhl.title.51784

[B44] Merkviladze MS Kvavadze ES (2002) List of ladybirds (Coleoptera, Coccinellidae) of Georgia. Proceedings of the Institute of Zoology, Tbilisi 21: 149–155.

[B45] Migeon A Arabuli T (2022) Rediscovery od *Serangiummontazerii* Fürsch in Georgia and updated list of the Coccinellidae of Georgia. Caucasiana 1: 1–6. 10.3897/caucasiana.1.e60966

[B46] Mittermeier RA Robles-Gil P Hoffmann M Pilgrim J Brooks T Mittermeier CG Lamoreux J Da Fonseca GAB (2004) Hotspots Revisited: Earth’s Biologically Richest and Most Endangered Terrestrial Ecoregions. CEMEX, Mexico City, 391 pp.

[B47] Mulsant E (1850) Species des Coléoptères trimères sécuripapes. Annales des Sciences Physiques et Naturelles d’Agriculture et d’Industrie (Deuxième Série) 2: xv + 1104 pp. 10.5962/bhl.title.8953

[B48] Mulsant E (1853) Supplément a la monographie des Coléoptères trimères sécuripapes. Annales de la Société Linnéenne de Lyon (nouvelle série) 1: 129–298. 10.5962/bhl.title.60609

[B49] Mulsant E (1866) Monographie des Coccinellides, I^er^ Partie – Coccinelliens. F. Savy / Deyrolle, Paris, 294 pp.

[B50] Mumladze L Japoshvili B Anderson EP (2020) Faunal biodiversity research in the Republic of Georgia: a short review of trends, gaps, and needs in the Caucasus biodiversity hotspot. Biologia 75: 1385–1397. 10.2478/s11756-019-00398-6

[B51] Myers N Mittermeier RA Mittermeier CG da Fonseca GAB Kent J (2000) Biodiversity hotspots for conservation priorities. Nature 403: 853–858. 10.1038/35002501 10706275

[B52] Radde G (1899) Die Sammlungen des kaukasischen Museums. Band I. Zoologie. Typographie der Kanzelei des Landeschefs, Tiflis, 520 pp.

[B53] Reitter E (1880) Bestimmungs-Tabellen der europäischen Coleopteren. I. Enthaltend die Familien: Cucujidae, Telmatophilidae, Tritomidae, Mycetaeidae, Endomychidae, Lyctidae und Sphindidae. Verhandlungen der kaiserlich-königlichen zoologisch-botanischen Gesellschaft in Wien 29: 71–100.

[B54] Reitter E (1890) Neue Coleopteren aus Europa, den angrenzenden Ländern und Sibirien, mit Bemerkungen über bekannte Arten. Zehnter Theil. Deutsche Entomologische Zeitschrift 1890, Heft 1: 165–176. 10.1002/mmnd.48018900324

[B55] Reitter E (1897) Dreissig neue Coleopteren aus russisch Asien und der Mongolei. Deutsche Entomologische Zeitschrift 1897, Heft 2: 209–228. 10.1002/mmnd.48018970115

[B56] Schneider O Leder H (1879) Beiträge zur Kenntniss der kaukasischen Käferfauna. Verhandlungen des naturforschenden Vereines in Brünn 17: 3–99.

[B57] Snegovaya N Zare Khormizi M (2022) Checklist of lady beetles (Coleoptera: Coccinellidae) in Azerbaijan Republic. Munis Entomology & Zoology 17: 1142–1154.

[B58] Szawaryn K Nedvěd O Biranvand A Czerwiński T Nattier R (2021) Revision of the genus *Coccidula* Kugelann (Coleoptera, Coccinellidae). ZooKeys 1043: 61–85. 10.3897/zookeys.1043.65829 34163295 PMC8213682

[B59] Tarkhnishvili D Gavashelishvili A Mumladze L (2012) Palaeoclimatic models help to understand current distribution of Caucasian forest species. Biological Journal of the Linnean Society 105: 231–248. 10.1111/j.1095-8312.2011.01788.x

[B60] Tobias VI (1975) A Review of the Braconidae (Hymenoptera) of the USSR. Amerind Publishing Co. Pvt. Ltd., New Delhi, viii + 164 pp.

[B61] Ukrainsky AS (2013) The multicoloured Asian lady beetle *Harmoniaaxyridis* Pall. (Coleoptera, Coccinellidae) in North Caucasus, Russia. Eurasian Entomological Journal 12: 35–38. [in Russian, with English abstract]

[B62] Victor T [Motschulsky VI] (1835) Description de quelques Coléoptères recueillis dans un voyage au Caucase et dans les provinces transcaucasiennnes russes en 1834 et 1835. Noveaux Mémoires de la Société Impériale des Naturalistes de Moscou 4: 313–323.

[B63] Victor T [Motschulsky VI] (1837) Description de quelques Coléoptères recueillis dans un voyage au Caucase et dans les provinces transcaucasiennnes russes en 1834 et 1835. Noveaux Mémoires de la Société Impériale des Naturalistes de Moscou 5: 413–425.

[B64] Wagner M (1852) Reise nach Persien und dem Lande der Kurden. Zweiter Band. Arnoldische Buchhandlung, Leipzig, iv + 315 pp.

[B65] Weise J (1879) Bestimmungs-Tabellen der europäischen Coleopteren. II. Coccinellidae. Zeitschrift für Entomologie (Neue Folge) 7: 88–156.

[B66] Weise J (1885) Bestimmungs-Tabellen der europäischen Coleopteren. II. Heft. Coccinellidae. II. Auflage. Mödling, 83 pp.

[B67] Weise J (1894) *Coelopterusarmeniacus*. Deutsche Entomologische Zeitschrift 1894: 144. 10.1002/mmnd.48018940549

[B68] Winkler A (1927) Catalogus Coleopterorum regionis palaearcticae. Pars 7. Albert Winkler, Wien, 753–880.

[B69] Zazanashvili N Manvelyan K Askerov E Mousavi M Krever V Kalem S Garforth M (2020) The boundaries and bio-physical features of the Caucasus ecoregion. In: Zazanashvili N Garforth M Bitsadze M (Eds) Ecoregional Conservation Plan for the Caucasus, 2020 Edition: Supplementary Reports.WWF, KfW, Tbilisi, 9–20.

